# Solid-to-solid polymorphic phase transitions in two isostructural Bi(III) complexes with 1-phenylethyl-*N*-ethylthiosemicarbazide and halogens

**DOI:** 10.1038/s41598-023-38561-4

**Published:** 2023-07-17

**Authors:** Anita M. Grześkiewicz, Grzegorz Dutkiewicz, Ozlem Aygun, Ibrahim I. Ozturk, Maciej Kubicki

**Affiliations:** 1 Faculty of Chemistry, Adam Mickiewicz University, Poznan, Poland; 2 Section of Inorganic Chemistry, Department of Chemistry, Tekirdag Namık Kemal University, 59030 Tekirdaǧ, Turkey

**Keywords:** Materials chemistry, Materials science, Condensed-matter physics, Structural materials, Techniques and instrumentation

## Abstract

Two isostructural (in room temperature) complexes of Bi(III) with halogens and sulfur ligands have been investigated in terms of the solid-to-solid phase transitions indicated by temperature. Both chloride and bromide (X) complexes of the general formula (µ_2_-X)-(BiX_2_L_2_)_2_ exhibit some phase transitions between 100 and 333 K, which, apart from the numerous similarities, show significant differences, which have been noted and analyzed in detail in this paper by using different techniques, i.e., powder and single crystal diffraction or DSC. The obtained results have also been collated with those obtained for solid solutions of both complexes.

## Introduction

Polymorphism, a widely occurring phenomenon of the existence of a given compound in different crystal forms, has attracted growing interest due to its scientific and industrial (for instance, in the pharmaceutical industry) importance. The existence of different polymorphic forms is generally related to different crystal packing and/or conformations; the differences can be large (for instance^1^) or quite tiny; the phase transformation between such forms can be, as a consequence, of a different nature and scope. Particularly interesting, from the viewpoint of understanding these phenomena, are the transformations that retain the crystallinity of the sample (single crystal-to-single crystal, SCSC transformations), thus allowing the use of diffraction techniques to monitor the changes or at least to compare the structures of different polymorphs. The comparison of both molecular and crystal structures can help in proposing the mechanism of a transformation and in estimating the roles of different factors, such as conformational changes and intermolecular interactions. These structural changes, as related to changing crystal structure without gaining a liquid phase, are rather minor, and such an analysis is often laborious and involves many better or worse justified hypotheses. It might also be noted that such reversible solid-to-solid phase transitions—although usually not polymorphic ones but involving, e.g., molecular change (solid-state reactions)—are becoming of great interest due to their possible application in thermal energy storage or data communication^2^. Phase transition materials (PTMs) usually display abrupt modifications of physical properties^3^ around the temperature of conversion and are considered functional materials, such as switchable and tunable dielectrics^3,4^, multiferroics^5^, and ferroelectrics^6^. Consequently, the field of interest in the case of PTM is very broad and covers inorganic^7,8^, metalloorganic^5,9,10^ and organic compounds^11–14^. Understanding the full mechanism of solid‒solid transitions and, consequently, the possibility of controlling the process of obtaining de facto different polymorphs is particularly important in the pharmaceutical industry mainly due to the possible difference in physicochemical or biological properties such as solubility, bioavailability or even activity, which also influences the formal and economic factors. The bismuth halides show semiconducting properties^15,16^ with non-toxic constituents, which make them even more interesting in terms of PTMs. Moreover, some of the halobismuthate(III) complexes have been successfully used to manufacture OFET-based memory devices^17^ and as polynuclear halide complexes of Bi(III) may display photochromism, thermochromism, luminescence or solvatochromism, they are subject of interest in design of new materials^18^.

**Figure 1:**
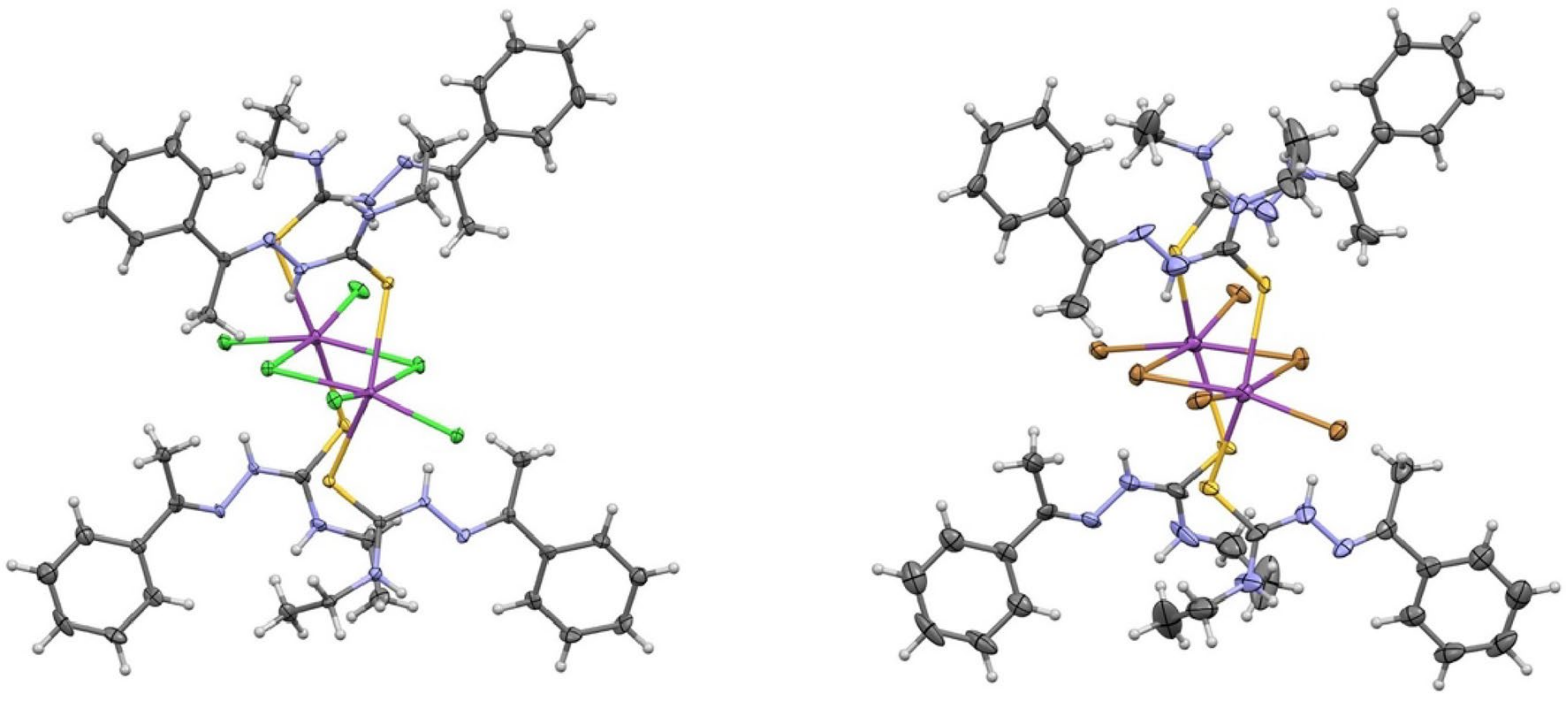
Perspective views of the complexes **1** (left) and **2** (right) as seen in the crystal structures at 100 K. The ellipsoids are drawn at the 50% probability level, hydrogen atoms are shown as fixed-size spheres. Only higher-occupied molecule of **2** is shown (cf. text).

In the course of our studies on bismuth(III) complexes with thiosemicarbazones, a remarkable group of N,S-donor ligands with a broad spectrum of pharmacological effects and versatile coordination behavior^19,20^, we obtained two isostructural complexes of general formulae (µ_2_-X)-(BiX_2_L_2_)_2_, where L is 1-phenylethyl-*N*-ethylthiosemicarbazide and X is Cl (**1**) or Br (**2**), cf. Fig. 1. The exchange of halogen ligands often leads to isostructural species, and for the special case of bismuth (III) and sulfur-coordinated ligands, such examples can be found in the CSD^21^, for instance, [(3,3′-methylenebis(1-isopropyl-1H-imidazole-2(3H)-thione))Bi(Cl/Br)_2_(μ_2_-Cl/Br)]_2_·CH3CN^22^ or Bi_5_(S_2_CNEt_2_)_8_(Br/Cl)_7_^23^; however, no peculiarities in the thermal characteristics of these compounds were observed. Interestingly, both **1** and **2** showed similar (but not identical) polymorphic phase transformations in the temperature range of 100–333 K. We present here a detailed investigation of these processes using a number of techniques and an attempt to rationalize the changes and differences in the behaviors of both compounds.

## Results

Figure 1 shows the perspective views of both complexes, as observed in their crystal structures at 100 K. The complexes are two-cantered, with pairs of halogen atoms in both bridging and terminal positions. Both Bi centers are 6-coordinated, with four halogen atoms and two sulfur atoms from two monodentate ligand molecules. The coordination geometries may be described as slightly deformed octahedral, with *trans* disposition of S atoms (Supplementary Material, Table S1).

The Bi-X bond lengths strongly depend on the halogen position; the bridging atoms are usually farther from the metallic centers than the terminal ones. For instance, at room temperature, the mean values for Bi-Br bond lengths are 2.72(1)/3.00(1) Å and for Bi–Cl 2.57(2)/2.86(2) Å, for terminal/*bridging* atoms, respectively. Similar trends are observed for all similar structures found in the CSD. The Bi–S distances are very similar in both room temperature structures (2.81(2) Å for (**1**) and 2.83(2) Å for (**2**)) and close to the typical values for this bond length (for 591 structures in the CSD, the mean value is 2.80(17) Å).

The crystal structures of both complexes show very similar patterns of intermolecular contacts, which—at least to some extent—determine the crystal packing modes. There are not only expected N–H···X hydrogen bonds but also weaker C–H···X and C–H···S interactions (Table S2).

Studies of the structures at different temperatures have shown that the two compounds are isostructural—as might be expected—but only at certain range of temperatures. Therefore, we decided to use some additional methods that proved to be useful in studying such phenomena, in particular powder X-ray diffraction (PXRD) and differential scanning calorimetry (DSC), which confirmed the presence of three different solid-state phases of complex **2** and four of **1** between 100 K and room temperature.

### Single crystal analysis

**Figure 2:**
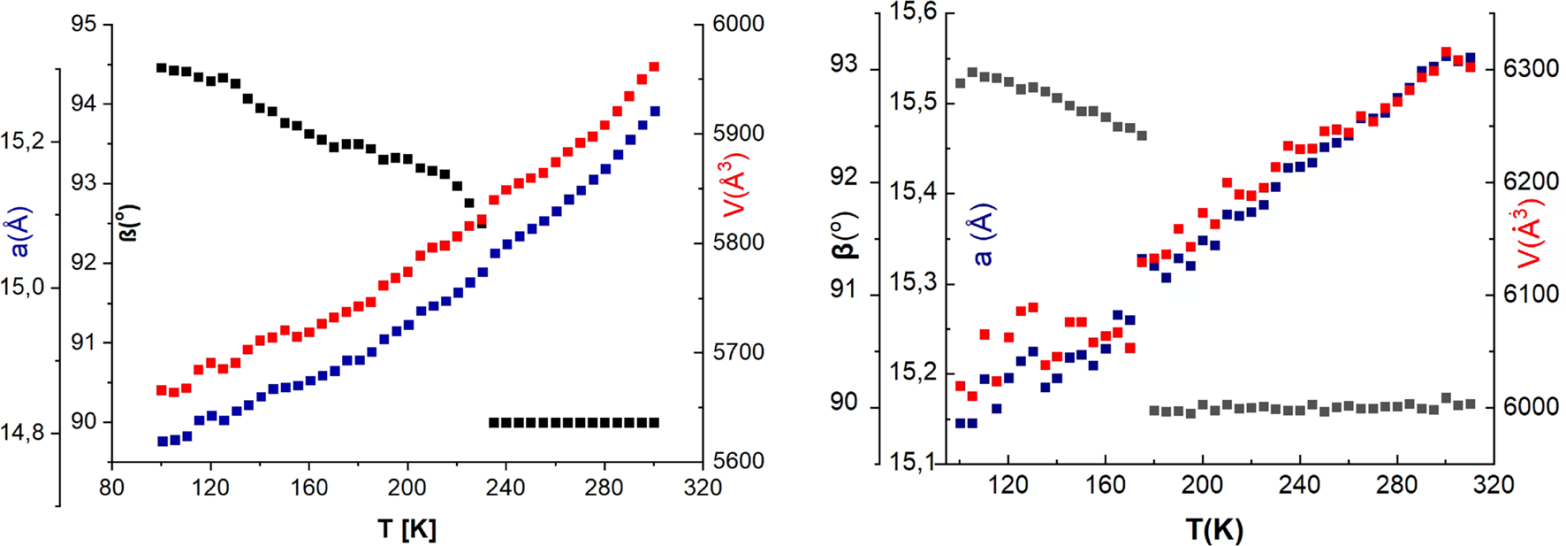
The plot of selected unit cell parameters of temperature (left—**1**, right—**2**) (blue—a parameter of the unit cell (Å), black—β angle (°), red—V, volume of the unit cell (Å^3^).

The unit cell parameters were obtained from the diffraction data using a standard least-squares procedure collected in the temperature range of 100–330 K with a step of 5 degrees. Figure 2 shows some of the unit cell parameters (a, β, V) as a function of temperature, and the full numerical data are included in the supplementary material (Table S3).

In both cases, the plots confirm the presence of a phase transition at approximately 230 K for **1** and approximately 175 K for **2**. For the low-temperature phase, a larger dispersion of the values is certainly observed, which is especially evident for **2**.

The structural changes in **1** and **2** are not very dramatic and certainly do not involve significant rearrangement of the molecular geometry or of the pattern of intermolecular interactions (Supplementary Material, Fig. S1); the structures are indeed relatively rigid at both the molecular and supramolecular levels. The differences between phases concern mostly the orientations of the phenyl rings and ethyl groups in the case of **1** and a slight displacement of the whole complex in the case of **2**. These seemingly subtle changes have quite significant consequences in terms of symmetry and induced twinning of the complexes.

Based on the PXRD and DSC results (vide infra), three structural changes were detected in the case of **1** (four phases) and two for Compound **2** (three phases, cf. Table 1). Consequently, full sets of X-ray diffraction data were collected for each phase, and attempts to find the most appropriate space groups to describe each phase were performed. This was neither an easy nor a straightforward task.

**Table 1:** R-factor parameters for refined structures in different space groups in different temperatures, but for the same crystal.

Temperature [K]	PO 1		PO 2	PO 3			
	Ccce	C2/c	P222_1_	C2/c	P2_1_/c	P2/c	
Complex 1							
333	**7.93**	6.68	4.64	8.84	8.28	8.90	Phase γ
303	**9.64**	7.55	6.07	25.85	–	–	Phase δ
RT	**7.95**	9.08	5.37	10.35	9.5	12.65	
240	29.39	**9.88**	17.77	32.13	31.35	34.27	Phase β
230	23.99	24.36	25.10	6.44	**4.0**	8.48	Phase α
220	22.05	22.67	22.17	8.91	**4.79**	**8.72**	
190	24.92	24.88	25.13	5.53	**2.91**	**8.08**	
100	27.97	27.15	–	6.41	**3.85**	11.09	
Complex 2							
333	**3.51**	3.90	7.84	8.32	8.11	8.49	Phase γ
303	**6.53**	6.69	7.66	7.97	7.71	7.7	
RT	**5.76**	7.78	6.88	7.21	6.96	7.05	
260	**6.12**	7.17	9.59	11.02	11.3	11.45	
240	–	**15.77**	13.94	25.12	24.62	25.88	Phase β
190	37.18	**18.32**	22.57	35.47	–	–	
170	25.68	23.6	–	–	–	–	Phase α
100	25.66	27.56	–	**6.41**	–	**5.51**	

*PO* Parameters orientation: for (1) :PO1 (a ≈ 16 Å, b ≈ 15 Å, c ≈ 23 Å, α, β, γ = 90°), PO2 (a ≈ 16 Å, b ≈ 23 Å, c ≈ 15 Å, α,β,γ = 90°), PO3 (a ≈ 15 Å, b ≈ 16 Å, c 23 Å, α, γ = 90° β ≈ 90°–92°), for (2) PO1 ( a≈ 17 Å, b ≈ 16 Å, c ≈ 23 Å, α, β, γ = 90°), PO2 (a ≈ 17 Å, b ≈ 23 Å, c ≈ 16 Å, α, β, γ = 90°–90.3°), PO3 (a ≈ 16 Å, b ≈ 17 Å, c 23 Å, α, γ = 90° β≈90°–92°).

Significant values are in bold.

While analyzing the symmetries of the crystals, a number of possible unit cell settings and space groups were found; the abridged list of these possibilities is presented in Table 1. In some cases, the choice of the appropriate space group was obvious, while in others, the alternative refinements led to very similar final descriptors and—of course—structures. In such cases, the criterion of higher symmetry was regarded as decisive (for instance, preference of Ccce over C2/c).

For both complexes, the lowest temperature phases **(α)** are twinned (in a nonmerohedral manner. cf. Fig. S2). which adds an additional level of complexity to the problem. Notably, the space Groups C2/c (PO1) and C2/c (PO3) have different symmetry directions (Table 1).

An analysis of Table 1 shows that almost all structures can be successfully refined in each of the selected groups (obviously with different quality). This confirms that the structural changes are truly subtle.

Nevertheless, the R-factor reaches the lowest values in the case of **1** for the P 2_1_/c space group for the lowest temperature phase and P 222_1_/Ccce in the higher temperature phase. For Compound **2,** the lowest temperature crystal structure is best described in monoclinic space groups; however, here, the most preferable ones are apparently P2/c or C2/c. Above 260 K, Ccce is definitely the best structure to describe Compound **2**. Consequently, for both Compounds **1** and **2**, the C 2/c (1) space group seems to be the intermediate phase; however, it is definitely distinguishable. It is also worth noting that for this phase (**β),** the unit cell values are close to the orthorhombic crystal system (β angle ≈90.0 in one crystal series measurement for **1** and ≈ 90.0–90.3 for **2**), which together with the rather poor refinement results may suggest that the β phase is actually a pseudomerohedral twin, which turns into a single crystal after transition to the γ phase (Ccce) for both **1** and **2.**

### Room temperature form

The crystals of complex **1** were kept for more than half a year at room temperature, and no signs of crystal decomposition were observed, which confirms the stability of the room-temperature γ form.

The crystal structures of complexes **1** and **2** at room temperature are highly isostructural (Table S4). However, differences are observable even in the diffraction patterns (Fig. S2), which in the case of **2** is typical for the C lattice, while **1** looks mixed and in some areas (at higher angles) perfectly meets the criteria of the C-lattice, while closer to the beam, it looks more like the P lattice.

As far as both complexes at room temperature can be solved and refined successfully, even though with varying quality (see Table 1) in the Ccce or P222_1_ space groups, in the diffraction pattern of **1** they are almost entirely, while the reflections that should be absent in the C lattice (h + k = 2n + 1) are only partially extinguished in the case of **2** (cf. Table S5). This may be related to the very small deviation from the higher symmetry in the case of **1**. The full systematic reflection extinction data are summarized in Table S5.

### Phase transitions

Both of the complexes at low temperature crystallize in the monoclinic system and show similar packing modes (cf. Fig. S1), but the observed discrepancies concern mostly the geometry of the complex molecule itself as well as the best-fitting space group.

For Compound **1,** the structure in the low-temperature phase, despite twinning, appears to be well defined. The compound crystallizes in the P2_1_/c space group. In the case of **2,** the transition to the new phase, in which twinning is observed, is clearly visible, but the choice of the most appropriate space group is not obvious. The beta angle has values slightly above 94°, which undoubtedly defines a monoclinic crystal system. Nevertheless, distinguishing between the C2/c (2) and P2/c space groups is not clear. Upon cooling, the symmetry changes from C2/c to P2/c. For 170 K (immediately after the phase transition), the intensities of reflections h + k = 2n + 1 are low, but they become larger as the temperature decreases to 100 K, where the structure can be solved and refined in both the P2/c and C2/c space groups with similar refinement quality. Additionally, the systematic reflection extinctions do not point clearly to one of the groups (Fig. S2). However, because the h + k = 2n + 1 reflexes are incompletely extinct, assuming that the more symmetrical group should be chosen, C2/c(2) is preferable.

The most significant structural change, which can be observed by means of single crystal X-ray diffraction, is related to the change in the diffraction pattern from single crystal (or possibly pseudomerohedral twin) to nonmerohedral twin (phase **β** to phase **α**). This phase transition can be seen between 230 and 235 K for **1** and 170 and 175 K for **2**. Both transitions are reversible and relatively abrupt. The layers of the reciprocal lattice illustrating this effect are shown in Fig. S3. The analysis of the structural effect for compound **2** is more demanding and probably slightly less accurate due to the poorer quality of the samples; however, it is still reliable.

At temperatures higher than room temperature for complex **1**, symmetry changes, as it becomes closer to Ccce. The degree of extinction of C-related reflections increases with increasing temperature, and for data collected at 333 K, it is much greater than for those at 303 K, at which point the transformation probably begins. However, in addition to the symmetry changes, it has also been observed that at 333 K, the crystal begins to split in a reversible manner. Interestingly, for complex **2**, which adopts Ccce symmetry at room temperature, none of these effects were detected (Fig. S2). Consequently, both crystals at 333 K are almost perfectly isostructural (isostructuality index^24^ 99.3%). Here, one should describe the room temperature structure of complex **1** as a separate phase or not, and the space group describing it should be determined. The DSC analysis indicates phase transformation above room temperature, the powder diffractograms are slightly but obviously different, and the single crystal refinement parameters change, which prompt us to conclude that for **1** between P2_1_/c and Ccce, there are two phases: **β** (monoclinic) and **δ** (orthorhombic), and the transformation between them is rather continuous than discrete. Even though the refinement quality (defined by R factors) seems to be better for space Group P222_1_ due to the increasing number of refinement parameters, the symmetry of the crystal still more closely resembles that of the Ccce space group, and consequently, this space group has been chosen. However, it should be considered that, due to partial extinction of the h + k = 2n + 1 reflections, neither the P222_1_ nor Ccce space group describes the delta phase more sufficiently than the other.

The phase transition from the room temperature phase to the **β** (monoclinic) phase in the case of Compound **2** can be easily observed from the analysis of the reflection extinction, which indicates the disappearance of glides perpendicular to the 010 and 001 directions (in unit cell 15/16/23—cf. Table S5) below 260 K, which indicates a structural change between 260 and 240, which can also be seen from the powder diffraction pattern (cf. PXRD section) and corresponds to the change from Ccce to the C2/c space group **(γ** to **β**). For Compound **1**, the **β** phase covers a smaller range of temperatures (240–260 K) than in the case of **2** (240–190 K) due to the closer phase transition to the **α** phase; however, it can still be detected both from the powder diffraction pattern as well as from the analysis of systematic extinctions and consequently from refinement parameters.

### Structure comparison

At room temperature, in the crystal structures of complexes **(1)** and **(2)**, bismuth atoms occupy a special position on the twofold axes in the [010] direction and c glide perpendicular to [100]. Moreover, Br1/Cl1 (bridging) atoms lie on the twofold axis in the [001] direction and n glide perpendicular to [010]. When the symmetry decreases to C2/c with decreasing temperature (beta phase), only bismuth atoms still occupy a special position (twofold axes in [010]), while in the alpha phase, neither bismuth nor halide atoms are present.

Figure S4 shows the result of the superimposition of complex **1** as determined in each phase. The structures overlap on the heavy atom cores. The most obvious outlier is the **α** phase, in which the ligands are significantly shifted relative to the other phases. The difference in the positions of the carbon atoms in the aromatic rings varies from approximately 0.57 to 1.26 Å.

Variations within the molecule of complex **1** for the remaining phases are much more subtle in comparison to **2**, which can also be seen in the very similar powder diffractograms (Fig. S4). The β phase is distinguished from the others by a disordered terminal chlorine atom (occupancy 0.65/0.35) (Fig. S4b), which was found only in this phase. Moreover, the analysis of the residual density maps in this phase indicates one more alternative orientation of the ligand, which, due to the apparently very small occupancy, cannot be properly modeled; however, the maxima of residual density maps in a number of independent measurements appear at the same positions, which may form a reasonable alternative orientation, thus strengthening the assumption that this may be an additional ligand position. It should be noted that these maxima disappear in both the **α** and **δ** phases. In turn, the atoms of the phenyl ring, although possessing relatively large displacement parameters, do not show the features of significant disorder, which is clearly visible for the **γ** and **δ** phases (Fig. S4c) and is actually the main discrepancy between those two phases. As shown in Fig. S4c, the mutual orientations of the disordered positions of the phenyl rings are different for those phases. Consequently, in most cases of complex **1,** the phase transitions can be described as order–disorder, with the exception of that from **δ** to **γ**, which seems to be closer to displacive.

In complex **2,** the differences between the phases are of a relatively different kind. Again, similarly disordered phenyl rings can be observed (Fig. S5c–e); however, due to the symmetry changes in the **α** and **β** phases within one complex molecule, there are two disordered phenyl rings and two ordered rings, while the **γ** phase is the best described by all four disordered phenyl rings. Consequently, the phase transition between the **γ** and **β** phases can be recognized as order–disorder, while that between **α** and **β** is rather displacive (Fig. S5b). Additionally, the occupancies of these two phenyl ring positions change upon transformation from **α** (≈ 0.5/0.5) to **β** and **γ** (≈ 0.3/0.7).

The similarity of complexes **1** and **2** is illustrated in Fig. S6. The greatest conformity achieves** γ** phases and the least achieves **α** phases, which has been confirmed by calculation of the isostructurality indices (Table S4). The isostructurality index has been calculated based on the higher-occupied positions of disordered groups, or if equivalent (**α**) exists for both disordered positions. The very high values of this parameter for high-temperature phases affirm that the actual discrepancies concern mainly the second position of the disordered part. Additionally, comparison of the unit cell data shows that they are very similar for these two compounds in the whole temperature range. The values oscillate at approximately 0.0175 (unit cell index) and 0.02 (elongation) (Fig. in the Supplementary Materials).

The high resemblance of the analyzed structures can also be observed in the fingerprint plots of the Hirshfeld surfaces (Fig. S7), although some differences can also be seen.

The most visible differences concern the short contacts between hydrogen atoms in the **α** phases; however, one should remember that the calculations are limited to nondisordered structures; thus, this effect is a consequence of not considering the other position rather than genuine contact, as the occupancy of both positions is equal.

For both complexes, the spokes corresponding to the Cl/Br···H contact are much smaller and more blurred in the case of the **β**,** γ**, and **δ** phases than for the **α** phase. This indicates the denser structure of the **α** phase in comparison with the others, which is also confirmed by the Kitaigorodskii packing index^25^ results (Table S6). The distributions of different contact types in the case of all phases are very similar, which is not surprising considering that packing modes in all structures are almost the same. The graph presenting the percentage of specific contacts is included in the Supplemental Material, Fig. S8.

### Powder XRD

Variable-temperature powder XRD studies were performed. For both compounds, diffractograms were recorded at temperatures within the range 130–310 K, with 10-degree steps in both heating and cooling procedures. Additionally, for Compound **2** in the range 166–203 K (suspected phase transition from **α** to **β** phase), the data were collected with a 3 degree step. The full diffractograms for both compounds are included in the supplementary materials.

The main discrepancies in the analyzed diffractograms concern the change in position or intensity of particular reflections. While some of these changes can be recognized as a consequence of changes in the unit cell parameters, the magnitude of a number of them and the coherence with the results of other methods used in this study allow us to find structure changing points.

**Figure 3:**
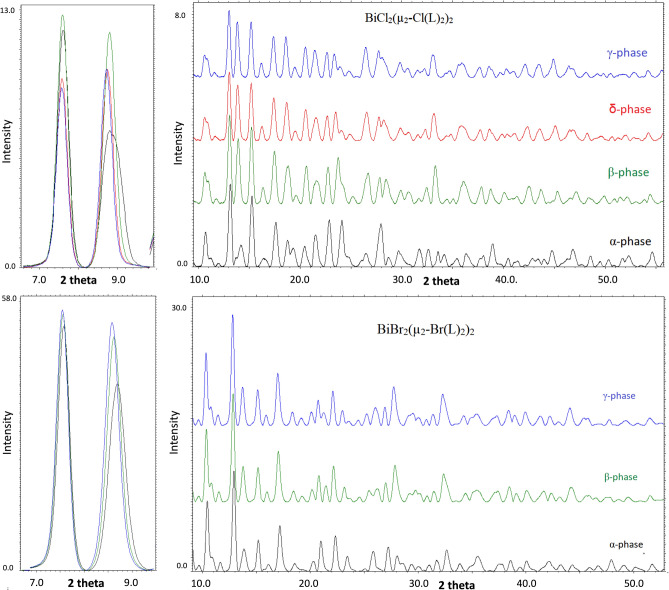
(upper panel) The selected ranges of powder X-ray diffractograms collected for complex **1**; (lower panel) Selected fragment of the temperature PXRD patterns for compound (**a**) (**2**), (**b**) (**1**) in first and last measurement cycle.

For Compound **1**, four phases have been recognized (cf. Fig. 3): (**α**) below 220 K, (**β**) between 230 and 260 K, (**δ**) approximately room temperature and **γ** above 300 K. The most significant differences in the diffraction pattern can be observed between phases **α** and **β,** which is related to the most significant changes in the structures (*cf.* single crystal results). The main differences between these two phases can be seen at approximately 2θ angles of 11°, 14°, 19°, 28° and 33.5°, which also refers to the theoretical diffractograms (Fig. SM). Dissimilarities between phases **β** and orthorhombic ones (**δ**,**γ**) are not that remarkable but are still substantial enough to classify them separately (2θ 19°, 24°, 28° and 33.5°). The most uncertain case is distinguishing between **δ** and **γ** phases. Actually, in the case of standard PXRD (not variable-temperature) and with no clear evidence of PT in that area from the DSC, it would be very difficult and would require very high resolution diffractograms because the differences in theoretical roentgenograms are very subtle. Nevertheless, we can observe dissimilarities at 2θ ≈ 27.5–28, which corresponds to the smaller angular differences between reflexes occurring in that area (from theoretical diffractograms; 0.3 for **δ**, 0.43 for **γ** phase, and 0.325/0.466 for the experimental), which can indicate a new phase. Figures showing the superimposed diffractograms of each group with the calculated diffractograms, based on the single crystal structures as well as full roentgenograms for all temperatures, have been included in the supplementary materials.

**Figure 4:**
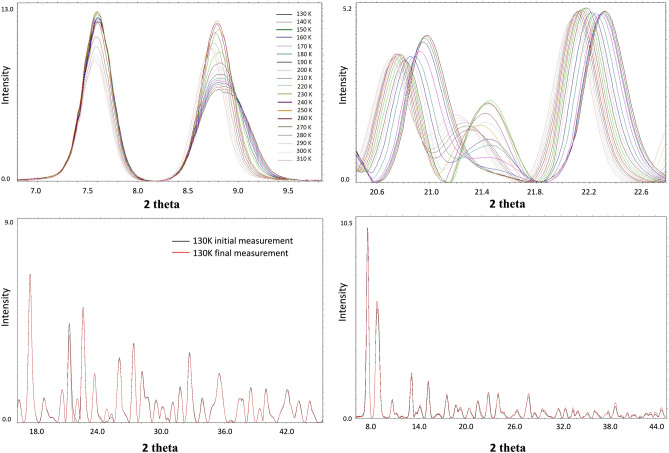
**4** (upper panel) The selected ranges of powder X-ray diffractograms collected for complex **1**; (lower panel) Selected fragment of the temperature PXRD patterns for compound (**a**) (**2**), (**b**) (**1**) in first and last measurement cycle.

The analysis of variable-temperature powder diffractograms allows for comparison not only of the positions of the peaks but also of their intensities, which can also provide information on possible structural transformations. As shown in Fig. 4, the intensity shifts within the presented peaks are more significant in some temperature ranges than in others. The analysis of a single reflex might not be very reliable; however, systematic observations for whole roentgenograms can be very informative. Consequently, abrupt changes in the increasing/decreasing trends of peak intensities can be regarded as confirmation of structural changes. For complex 1, such a change is observed between 210 and 230 K, which corresponds to the α to β PT and between 260 and 280 K (β to δ). In fact, even the δ to γ PT can be observed for some reflections (for instance, 2θ = 8.7°, 18.55°, 20.5°, 21.45°, 22.6°, etc.; Fig. S9 in the SM).

**Figure 5:**
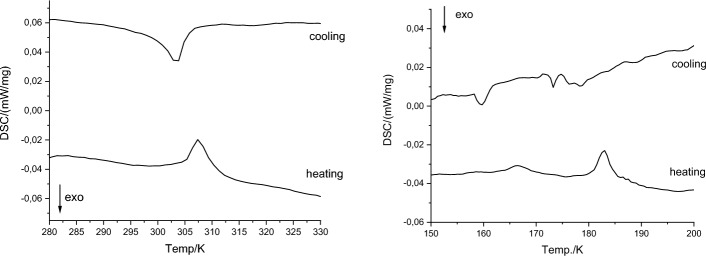
DSC curves for complex (**a**) **1**, (**b**) **2** in heating and cooling cycles.

A similar analysis was performed for Compound **2,** and only three phases (**α**, **β**, **γ**) were detected (Fig. S10). Analogous to complex **1,** the main differences are observable between phase **α** and others; however, they are even smaller than in complex **1**, which indicates fewer structural changes between those phases in complex **2**. The most significant changes concern reflection 133 (Fig. 4, Supplementary Fig. S10) (2θ ca. 21.25°–21.45°), which in the **γ** phase shifts to lower angles (2θ ≈ 21.25°) toward the **β** phase (2θ ≈ 21.45°) and is extinct in the** α** phase (2θ ≈ 21.45°). The other important area of comparison is 2θ = 25.8°, where in the **α** phase, a single peak can be observable, while in the **β** and **γ** phases, there is a saddle point between two other peaks. The other interesting feature is that the curves for 180 and 190 K seem to represent an intermediate state between the **β** and **α** phases, which may suggest that symmetry changes before twinning occurs (approximately at 175 K). This conclusion is supported by DSC analysis, where we can see two energetic effects in the 20 K range (Fig. 5).

Distinguishing between the **β** and **γ** phases is also problematic due to the very subtle changes in the structure, but even in this case, a difference can be observed at 2 theta at approximately 21.4° and 29.5°, which are also the most significant discrepancies in the theoretical diffractograms (Fig. S9). It should be noted again that taking into account such subtle differences in diffractograms is only reasonable when analysis concerns temperature variable measurements for a single sample taken in a single measuring series or with very good resolution.

Interestingly, the diffractograms for Compound **2** for the heating and cooling regimes are not exactly the same. They fully overlap at temperatures above 220 K; however, at 210 K, small discrepancies near 2θ angles of 21.5° and 24.5° can be observed (Fig. S11); this apparently very subtle effect increases with decreasing temperature and results in the appearance of additional peaks at 21.75° and 24.6° in comparison to the first measurement in the cycle. This suggests that during slow cooling, not all of the **β** phase is transferred to the **α** phase, and consequently, the direct transfer from the **γ** to α phase is more efficient than that through the intermediate **β** phase.

It should be stressed that no similar effect was observed in the case of Compound **1**.

### TG/DSC

The DSC curves for both complexes are presented in Fig. 5. The thermal effects in both complexes occur in different ranges but are always related to one of the PTs. Because the thermal effects related to the structural changes are very small, DSC analysis was performed a few times to exclude a measurement disturbance. The full DSC data are enclosed in the supplementary materials.

For Compound **1**, one small endothermic peak occurs at approximately 307 K upon heating, which corresponds to the exothermic peak at approximately 304 K upon cooling (revealing a reversible phase transition with a relatively small thermal hysteresis of ~ 3 K, ΔH ≈ 0.24 J/g). This effect is probably related to the **γ** to **δ** phase transition. This subtle thermal effect is reversible, but only if the melting point is not reached. The transformation to the liquid phase results in obtaining a glass phase during cooling in both complexes (c.a. 322 K (**1**) and 328 K (**2**) on cooling, 328 K (**1**) and 335 K (**2)** on heating).

For Compound **2,** two subtle endothermic (on heating) peaks at 183 K and 167 K occur, which are related to two exothermic signals at 180–173 K (because the signal is very irregular and defining its maximum is rather difficult, we decided to use range of temperatures instead of single value) and 160 K while cooling. Based on the ΔH associated with those signals, we can conclude that the peak at 167 K corresponds to 160 K (hysteresis 7 K, ΔH ~ 0.07 J/g). Consequently, the remaining peaks are also related (ΔH ~ 0.17 J/g). As previously mentioned, these two effects may be associated with two structural changes: one in which a crystal from the **β** phase starts to transfer to the **α** phase and the second related to changing a pseudomerohedral/single crystal phase into a nonmerohedral phase.

Surprisingly, although we observed similar conversion from the **α** to** β** phase on both single crystal and powder X-ray diffraction analysis for Compound **1**, this phase transition has not been recorded on the DSC curves for **1**; thus, this conversion, in addition to larger structural changes, seems to require less energy than in the case of **2**.

### Solid solutions

Because the crystal structures are very similar at all temperatures, attempts to obtain and characterize the solid solution of the analyzed complexes were performed. Considering that at room temperature, a more reliable model was found for complex **2,** a solid solution with a predominance of bromide was prepared. Based on the powder X-ray analysis (see below) in the temperature range of 100–310 K, three phases were established, and for each phase, single-crystal X-ray diffraction data were collected. Consequently, at room temperature, the **γ** phase was observed, at 220 K, the** β** phase was observed, and at 100 K, two phases were separated: a definitely dominant **β** phase and a minor but still visible **α** phase.

The structures of all phases of the solid solution have been determined and refined with an approximately 30/70 ratio of chlorine to bromine in bridged halogen atoms and 26/74 in terminal halogen atoms. The influence of the chloride fraction on the bromide structure was already apparent during crystallization and resulted in much better quality of the obtained crystals. The crystals were definitely bigger and more regular than for the pure bromine complex (**2**), and the chlorine addition also results in better refinement parameters for both **α** and **β** forms at a temperature greater than 180 K (Table S5).

In all cases, chlorine and bromine atoms occupy the same position in the asymmetric part of the unit cell.

### PXRD for solid solution

**Figure 6:**
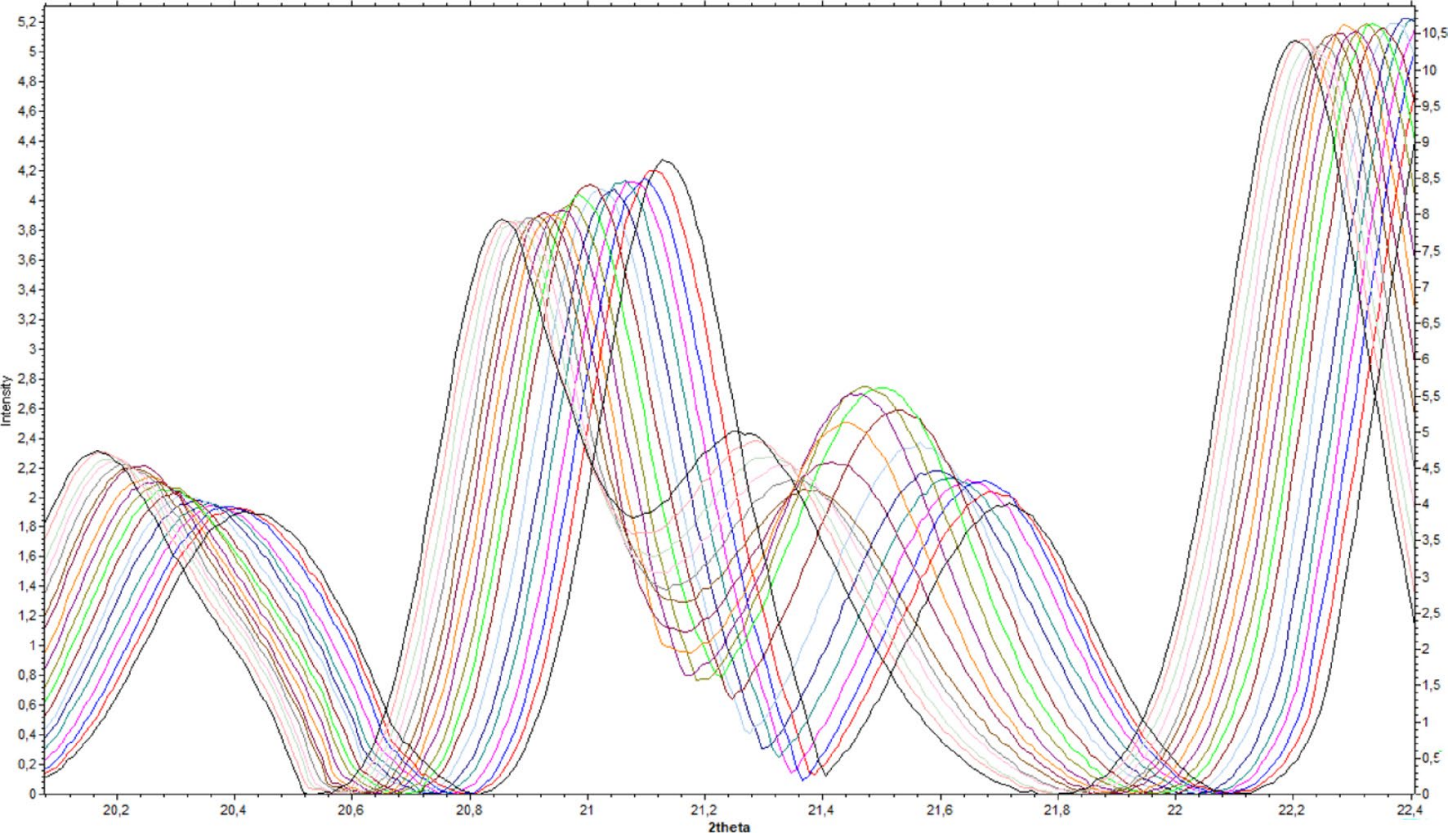
The powder X-ray diffractograms collected for the solid solution sample.

Powder X-ray data were collected for the solid solution sample and compared with the pure forms of complexes **1** and **2** (Fig. 6). As expected, the signals from reflections for the solid solution are between those from pure forms, more resembling and shifted toward the signals from complex **2**.

By analogy with complexes **1** and **2,** variable-temperature powder XRD studies were performed, and effects similar to those observed for the pure forms were expected. However, the analysis of the PXRD patterns shows quite regular intensity and position changes in most reflections. Fortunately, for the “strategic” reflections, continuous change of their position is disturbed. Figure 6 presents the apparently most informative part of the roentgenogram, which allows the identification of the different phases. Remarkably, no pure alpha phase is visible here, although three different areas can be detected: 130–180 K, 190–250 K and > 260 K.

As we have established (based on the monocrystal studies), the first range corresponds to the** γ** phase, the second to the **β** phase and the third to the split crystal with dominant **β** and minor **α** phases, which can be observed in the powder diffractogram as a reduction in the intensity of the signal at 2θ ≈ 21.65°. Thus, the solid solution that has formed seems to favor the** β** form instead of the **α** form.

## Experimental

### Synthesis

#### Complex 1

The bismuth(III) chloride (158 mg, 0.5 mmol) was dissolved in 10 mL methanol. Then, 10 mL methanolic solution of acetophenone-*N*-ethly-thiosemicarbazone was added (221 mg, 1.0 mmol). The yellow solution was refluxed for 4 h. The yellow crystals were collected from slow evaporation of the solution at room temperature. The crystals were washed with a small amount of cold methanol and dried in vacuo.

#### Complex 2

The bismuth(III) bromide (224 mg, 0.5 mmol) was dissolved in 10 mL methanol. Then, 10 mL methanolic solution of acetophenone-*N*-ethly-thiosemicarbazone was added (221 mg, 1.0 mmol). The yellow solution was refluxed for 4 h. The yellow crystals were collected from slow evaporation of the solution at room temperature. The crystals were washed with a small amount of cold methanol and dried in vacuo.

#### Solid solution

The bismuth(III) bromide (224 mg, 0.5 mmol) was dissolved in 10 mL methanol and a few drops of HCl were added. Then, 10 mL methanolic solution of acetophenone-*N*-ethly-thiosemicarbazone was added (332 mg, 1.5 mmol). The yellow solution was refluxed for 4 h. The yellow crystals were collected from slow evaporation of the solution at room temperature. The crystals were washed with a small amount of cold methanol and dried in vacuo.

## Methods

### DSC

The DSC analysis has been performed DSC214 Polyma (NETZSCH,Selb,Germany) under the nitrogen atmosphere heating rate approximately 10°/min. The measurement started from room temperature and samples were first cool down the temperature of 113.15 K than rise up to the 373.15 K and again cool down to the room temperature. As far as the low temperature effect could be observed only for the complex **2** the low temperature measurement has been repeated solely to this sample. In case of complex **1,** the measurement repetition has been performed to the 273.15 K.

### PXRD

The variable-temperature powder X ray studies has been performed with Agilent Technologies SuperNova diffractometer with monochromated CuK_α_ radiation (λ = 1.54178 Å) For both compounds diffractograms were recorded at temperatures in the range 130–310 K, with 10-degree steps in both heating and cooling procedures. Additionally, for the compound **2** in the range166–203 K (suspected phase transition from α phase to β phase the data were collected with 3 degrees step. The 2θ range for the measurement was 0°–50° with 30 s exposure time. Diffractograms was analysed with KDif v.3.01b program from the Kalvados package^26^.

### Single crystal X-ray measurement

X-ray diffraction data were collected by the ω-scan technique on a four-circle Rigaku Xcalibur diffractometer equipped with an Eos detector equipped with a graphite-monochromized MoKα radiation source (λ = 0.71073 Å)^27^. The data were corrected for Lorentz-polarization effects as well as for absorption^28^. Accurate unit-cell parameters were determined by a least-squares fit of the reflections of the highest intensity, which were chosen from the whole experiment. The calculations were mainly performed within the OLEX2^29^ and WinGX program system^30^. The structures were solved with ShelxT^31^ and refined with the full-matrix least-squares procedure on F2 by SHELXL-2015/2017^32^. Scattering factors incorporated in SHELXL were used. All non-hydrogen atoms were refined anisotropically, and hydrogen atoms were located at the calculated positions and refined as a “riding model” with isotropic thermal parameters fixed at 1.2 times the Ueq of the appropriate carrier atom for compounds Hirshfeld surfaces/fingerprints have been established using CrystalExplorer software^33^. The Kitaigorodskii packing index has been calculated using Platon software^34^.

Most relevant crystallographic parameters have been summarized in Table S5.

### Summary

**Figure 7:**
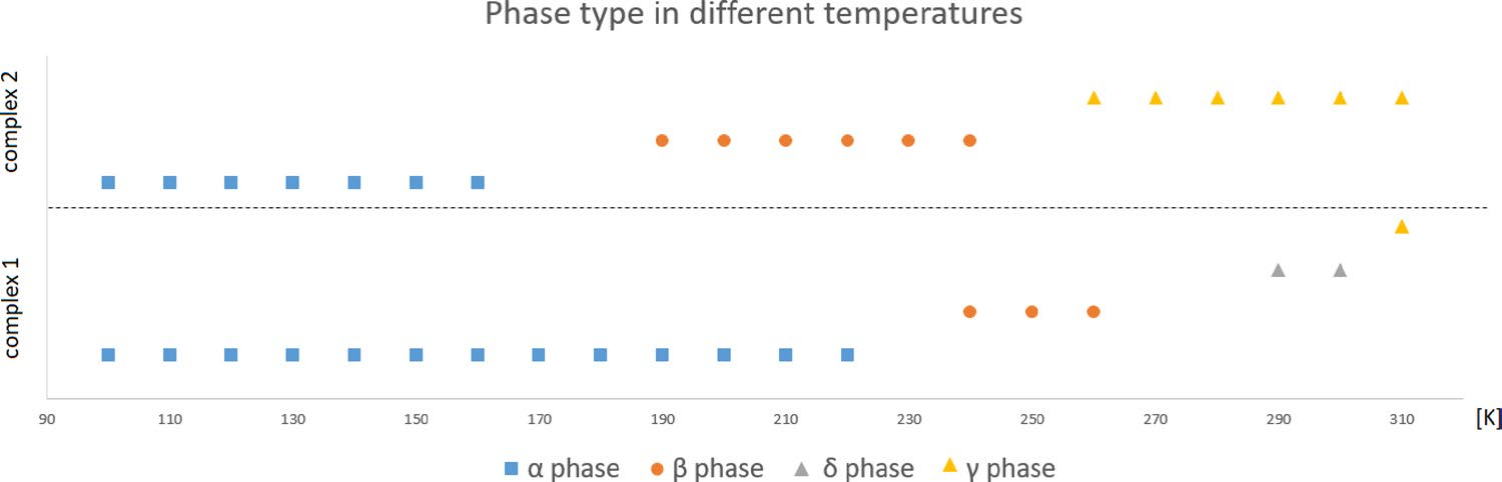
A collection of the information about different phases in compounds **1** and **2**.

In this study, four phases were found in the case of complex **1,** and three phases were found for complex **2** within the 100–333 K temperature range. The recognized phase transitions are mostly order–disorder- or displacive-type with the occurrence of secondary (annealing) twinning in the process of **β → α** conversion. In the case of both complexes, the lowest temperature **α** phase structures are characterized by the lowest but still high structural similarity (≈ 97.5%). The highest structural resemblance was found for the **γ** phases (99.3%). Despite the great similarity of structures from established phases, the phase transition occurs at a different temperature for complex **1** (**α → β** 220–240 K, **β → δ** 270–290 K **δ → γ** ≥ 310 K) than complex **2** (**α → β** 160–190 K, **β → δ** 240–260 K). Consequently, the analog phases appear in very narrow temperature ranges (Fig. 7).

Comparing the results for complexes **1** and **2,** the corresponding transitions are shifted for **1** to higher temperatures and are more continuous. The phase type for complex **1** is rather constant for given temperatures, and for complex **2,** it depends on the path the crystal has taken to reach a given temperature. Thus, during the fast cooling, at low temperature, only the form **α** has been obtained, while in a temperature reduction taking place over a longer period, the **β** form can also be recognized, at least from the powder pattern.

The addition of chloride to complex **2** results in the formation of a solid solution of **1** and **2**, which also influences the phase transitions of the mentioned compounds. The main difference concerns the **β(γ) → α** phase transition, which occurs at a similar temperature to complex **2** with visible splitting. In this case, there is no twinning, but two crystal forms occur: the dominant one, of the **β** phase, and definitely minor, of the **α** phase. As the **α** form represents only a few percent of the partitioned crystal, it may be inferred that the **β** form is stabilized by the addition of chlorine to the crystal of complex** 2**. The **β** form covers a wider temperature range in complex **2** than in complex 1, and it seems to be quite reasonable.

## Supplementary Information

Supplementary Information.

## Data Availability

The datasets used and/or analysed during the current study available from the corresponding author on reasonable request.
