# Supplementary material for: Solid-to-solid polymorphic phase transitions in two isostructural Bi(III) complexes with 1-phenylethyl-N-ethylthiosemicarbazide and halogens
Source: Sci Rep. 2023 Jul 17;13:11487. doi: 10.1038/s41598-023-38561-4 (PMC10352232; doi:10.1038/s41598-023-38561-4)
Supplement: Supplementary file 1 — Supplementary Information. [file 41598_2023_38561_MOESM1_ESM.zip › Supplementary materials.docx]

**Supplementary materials**

1. **Table S1**. Selected geometrical parameters for studied compounds
2. **Table S2**. Geometry of the intermolecular contacts in **1** and **2** (in italics) in **γ** phase (Å, º)
3. **Table S3**. Unit cell parameters in different temperatures
4. **Table S4.** Isostructurality index calculated for analogues phases
5. **Table S5**. The systematic analysis of reflection extinction in different temperatures also indicates some structural and symmetry changes.
6. **Table S7**. Relevant crystallographic and refinement data
7. **Table S6.** Kitaigorodskii packing index (%) calculated for more occupied position of disordered structures
8. **Figure S1.** The crystal packing along [001] direction for compounds **1** (left) and **2** (right) at 100K (lower panel) and at room temperature (upper panel)
9. **Figure S2.** Ewald representation along [001]for complexes **1** (top) and **2** (bottom) at different temperatures.
10. **Figure S3.** The layers of crystal a) **1** and b) **2** in temperatures close to the phase transition from phase 2 to phase 1
11. **Figure S4**. a) The superposition of complex **1** molecules from different phases
12. **Figure S5** a),b) The superposition of complex **2** molecules from different phases (**α**- black **β-**green **γ-** light blue from different views) Orientation of disordered phenyl rings in c) **α**, d) **β** e) **γ** phases.
13. **Figure S6**. Molecular overlay of the a) **α**, b) **β** c) **γ** phases of complexes **1** (green) and **2** (purple)
14. **Figure S7.** Hirshfeld fingerplots of complex molecules in different phases.
15. **Figure S8**. The unit cell similarity index and elongation vs. temperature [K]
16. **Figure S9.1.** Theoretically generated powder pattern for complex **1**:
17. **Figure S9.2.** Theoretically generated powder pattern for complex **2**.
18. **Figure S10.** The powder X-Ray diffractogrmas collected for 130 K black, 240 K green, 290K blue one form complex **2**
19. **Figure S11.** Selected fragment of the temperature PXRD patterns for compound **2** at four temperatures: red – first measurement in the cycle, black -second measurement in the cycle.
20. **Figure S12.** Fragments of the temperature PXRD patterns for compound **2** at different temperatures

**Table S1**. Selected geometrical parameters for studied compounds

Complex **1**

(α-phase)

Bi2—Cl4 2.8116 (19) Bi1—Cl4 2.9234 (18)

Bi2—Cl3 2.8126 (17) Bi1—Cl3 2.9337 (19)

Bi2—Cl6 2.6075 (18) Bi1—Cl1 2.5575 (18)

Bi2—Cl5 2.5978 (19) Bi1—Cl2 2.539 (2)

Bi2—S70 2.8527 (18) Bi1—S30 2.827 (2)

Bi2—S50 2.8073 (18) Bi1—S10 2.827 (2)

Cl4—Bi2—Cl3 95.33 (5) Cl1—Bi1—Cl4 162.44 (6)

Cl4—Bi2—S70 69.86 (5) Cl1—Bi1—Cl3 89.11 (6)

Cl3—Bi2—S70 85.81 (5) Cl1—Bi1—S30 80.54 (6)

Cl6—Bi2—Cl4 88.00 (6) Cl1—Bi1—S10 88.48 (6)

Cl6—Bi2—Cl3 168.12 (6) Cl2—Bi1—Cl4 88.89 (6)

Cl6—Bi2—S70 106.02 (6) Cl2—Bi1—Cl3 166.38 (6)

Cl6—Bi2—S50 96.62 (6) Cl2—Bi1—Cl1 95.58 (7)

Cl5—Bi2—Cl4 167.05 (6) Cl2—Bi1—S30 88.90 (6)

Cl5—Bi2—Cl3 88.99 (6) Cl2—Bi1—S10 81.66 (6)

Cl5—Bi2—Cl6 90.24 (6) S30—Bi1—Cl4 116.60 (5)

Cl5—Bi2—S70 98.38 (6) S30—Bi1—Cl3 79.25 (6)

Cl5—Bi2—S50 104.67 (6) S10—Bi1—Cl4 75.35 (5)

S50—Bi2—Cl4 88.28 (5) S10—Bi1—Cl3 111.31 (6)

S50—Bi2—Cl3 72.14 (5) S10—Bi1—S30 164.77 (6)

S50—Bi2—S70 147.47 (5) Bi2—Cl4—Bi1 87.22 (5)

Cl4—Bi1—Cl3 90.44 (5) Bi2—Cl3—Bi1 87.01 (5)

(β-phase)

Bi2—Cl2 2.853 (4) Bi1—Cl2^i^ 2.879 (4)

Bi2—Cl3 2.603 (8) Bi1—Cl1^i^ 2.565 (8)

Bi2—Cl3^i^ 2.603 (8) Bi1—Cl1 2.565 (8)

Bi2—Cl3A^i^ 2.560 (10) Bi1—Cl1A^i^ 2.557 (10)

Bi2—Cl3A 2.560 (10) Bi1—Cl1A 2.557 (10)

Bi2—S2^i^ 2.797 (12) Bi1—S1 2.829 (12)

Bi2—S2 2.797 (12) Bi1—S1^i^ 2.829 (12)

Bi2—S6 2.831 (13) Bi1—S10^i^ 2.838 (14)

Bi2—S6^i^ 2.831 (13) Bi1—S10 2.838 (14)

Bi1—Cl2 2.879 (4)

Cl2—Bi2—Cl2^i^ 91.91 (18) S6—Bi2—S6^i^ 164.5 (10)

Cl3^i^—Bi2—Cl2^i^ 90.1 (2) Cl2^i^—Bi1—Cl2 90.87 (17)

Cl3—Bi2—Cl2^i^ 165.61 (19) Cl1^i^—Bi1—Cl2^i^ 89.4 (2)

Cl3—Bi2—Cl2 90.1 (2) Cl1—Bi1—Cl2 89.4 (2)

Cl3^i^—Bi2—Cl2 165.61 (19) Cl1^i^—Bi1—Cl2 166.5 (2)

Cl3^i^—Bi2—Cl3 91.6 (4) Cl1—Bi1—Cl2^i^ 166.5 (2)

Cl3^i^—Bi2—S2 101.7 (4) Cl1—Bi1—Cl1^i^ 93.5 (4)

Cl3—Bi2—S2 96.4 (3) Cl1—Bi1—S10^i^ 84.2 (4)

Cl3^i^—Bi2—S2^i^ 96.4 (3) Cl1^i^—Bi1—S10 84.2 (4)

Cl3—Bi2—S2^i^ 101.7 (4) Cl1—Bi1—S10 85.6 (5)

Cl3A^i^—Bi2—Cl2 165.5 (3) Cl1^i^—Bi1—S10^i^ 85.6 (5)

Cl3A^i^—Bi2—Cl2^i^ 90.1 (3) Cl1A^i^—Bi1—Cl2^i^ 90.0 (3)

Cl3A—Bi2—Cl2 90.1 (3) Cl1A—Bi1—Cl2^i^ 167.1 (3)

Cl3A—Bi2—Cl2^i^ 165.5 (3) Cl1A—Bi1—Cl2 90.0 (3)

Cl3A^i^—Bi2—Cl3A 91.5 (5) Cl1A^i^—Bi1—Cl2 167.1 (3)

Cl3A—Bi2—S6^i^ 56.7 (4) Cl1A—Bi1—Cl1A^i^ 92.1 (5)

Cl3A—Bi2—S6 111.1 (5) S1^i^—Bi1—Cl2^i^ 91.7 (3)

Cl3A^i^—Bi2—S6 56.7 (4) S1—Bi1—Cl2 91.7 (3)

Cl3A^i^—Bi2—S6^i^ 111.1 (5) S1^i^—Bi1—Cl2 68.2 (3)

S2—Bi2—Cl2 92.3 (3) S1—Bi1—Cl2^i^ 68.2 (3)

S2^i^—Bi2—Cl2 69.3 (3) S1—Bi1—S1^i^ 151.9 (8)

S2—Bi2—Cl2^i^ 69.3 (3) S10^i^—Bi1—Cl2^i^ 109.1 (4)

S2^i^—Bi2—Cl2^i^ 92.3 (3) S10—Bi1—Cl2 109.1 (4)

S2—Bi2—S2^i^ 154.0 (8) S10—Bi1—Cl2^i^ 81.6 (5)

S6^i^—Bi2—Cl2 81.7 (4) S10^i^—Bi1—Cl2 81.6 (5)

S6—Bi2—Cl2^i^ 81.7 (4) S10—Bi1—S10^i^ 165.2 (10)

S6^i^—Bi2—Cl2^i^ 109.4 (4) Bi2—Cl2—Bi1 88.61 (10)

S6—Bi2—Cl2 109.4 (4)

Symmetry code(s): (i) −*x*, *y*, −*z*+1/2.

(δ -phase)

Bi1—Cl1^i^ 2.571 (4) Bi1—S1^i^ 2.831 (8)

Bi1—Cl1 2.571 (4) Bi1—S1A 2.827 (15)

Bi1—Cl2 2.868 (3) Bi1—S1A^i^ 2.827 (15)

Bi1—Cl2^ii^ 2.868 (3) Cl2—Bi1^iii^ 2.868 (3)

Bi1—S1 2.831 (8)

Cl1^i^—Bi1—Cl1 92.4 (2) Cl2—Bi1—Cl2^ii^ 90.89 (12)

Cl1^i^—Bi1—Cl2 89.98 (12) S1^i^—Bi1—Cl2^ii^ 93.0 (6)

Cl1^i^—Bi1—Cl2^ii^ 166.30 (8) S1^i^—Bi1—Cl2 69.6 (4)

Cl1—Bi1—Cl2 166.30 (8) S1—Bi1—Cl2 93.0 (6)

Cl1—Bi1—Cl2^ii^ 89.98 (12) S1—Bi1—Cl2^ii^ 69.6 (4)

Cl1—Bi1—S1^i^ 96.7 (4) S1—Bi1—S1^i^ 155.7 (14)

Cl1—Bi1—S1 100.1 (6) S1A^i^—Bi1—Cl2^ii^ 107.6 (10)

Cl1^i^—Bi1—S1^i^ 100.1 (6) S1A—Bi1—Cl2^ii^ 79.8 (8)

Cl1^i^—Bi1—S1 96.7 (4) S1A—Bi1—Cl2 107.6 (10)

Cl1^i^—Bi1—S1A 86.9 (7) S1A^i^—Bi1—Cl2 79.8 (8)

Cl1—Bi1—S1A 86.0 (10) S1A—Bi1—S1A^i^ 170 (2)

Cl1^i^—Bi1—S1A^i^ 86.0 (10) Bi1^iii^—Cl2—Bi1 89.11 (12)

Cl1—Bi1—S1A^i^ 86.9 (7)

Symmetry code(s): (i) *x*, -*y*+3/2, -*z*+3/2; (ii) -*x*+1, *y*, -*z*+3/2; (iii) -*x*+1, -*y*+3/2, *z*.

(γ-phase)

Bi1—Cl1 2.863 (3) Bi1—S10^i^ 2.827 (6)

Bi1—Cl1^i^ 2.863 (3) Bi1—S10A 2.87 (2)

Bi1—Cl2 2.557 (3) Bi1—S10A^i^ 2.87 (2)

Bi1—Cl2^i^ 2.557 (3) Cl1—Bi1^ii^ 2.863 (3)

Bi1—S10 2.827 (6)

Cl1^i^—Bi1—Cl1 90.37 (12) Cl2^i^—Bi1—S10^i^ 97.50 (18)

Cl1^i^—Bi1—S10A 108.9 (4) Cl2—Bi1—S10A 84.4 (4)

Cl1—Bi1—S10A 82.2 (4) Cl2—Bi1—S10A^i^ 85.0 (4)

Cl1—Bi1—S10A^i^ 108.9 (4) Cl2^i^—Bi1—S10A 85.0 (4)

Cl1^i^—Bi1—S10A^i^ 82.2 (4) Cl2^i^—Bi1—S10A^i^ 84.4 (4)

Cl2—Bi1—Cl1 89.85 (11) S10^i^—Bi1—Cl1^i^ 70.93 (15)

Cl2^i^—Bi1—Cl1 166.61 (8) S10—Bi1—Cl1 70.93 (15)

Cl2—Bi1—Cl1^i^ 166.61 (8) S10—Bi1—Cl1^i^ 95.22 (16)

Cl2^i^—Bi1—Cl1^i^ 89.85 (11) S10^i^—Bi1—Cl1 95.22 (16)

Cl2—Bi1—Cl2^i^ 93.0 (2) S10—Bi1—S10^i^ 160.7 (4)

Cl2—Bi1—S10 97.50 (18) S10A^i^—Bi1—S10A 164.7 (9)

Cl2^i^—Bi1—S10 95.72 (17) Bi1^ii^—Cl1—Bi1 89.63 (12)

Cl2—Bi1—S10^i^ 95.72 (17)

Symmetry code(s): (i) −*x*+1, *y*, −*z*+1/2; (ii) −*x*+1, −*y*+3/2, *z*.

Complex **2**

(α-phase )

Bi1—Br1^i^ 2.9753 (12) Bi1—S30A 2.953 (15)

Bi1—Br1 3.0158 (12) Bi1—S10 2.785 (6)

Bi1—Br2 2.7354 (13) Bi1—S10A 2.928 (14)

Bi1—Br3 2.7118 (13) Br1—Bi1^i^ 2.9753 (12)

Bi1—S30 2.785 (6)

Br1^i^—Bi1—Br1 93.49 (3) Br3—Bi1—S10A 87.9 (3)

Br2—Bi1—Br1 88.47 (4) S30—Bi1—Br1^i^ 71.81 (15)

Br2—Bi1—Br1^i^ 168.19 (4) S30—Bi1—Br1 93.72 (13)

Br2—Bi1—S30 96.45 (15) S30—Bi1—S10 157.52 (19)

Br2—Bi1—S30A 89.8 (3) S30A—Bi1—Br1 107.5 (3)

Br2—Bi1—S10 99.48 (13) S30A—Bi1—Br1^i^ 78.5 (3)

Br2—Bi1—S10A 83.8 (3) S10—Bi1—Br1 71.07 (14)

Br3—Bi1—Br1^i^ 89.45 (4) S10—Bi1—Br1^i^ 92.17 (14)

Br3—Bi1—Br1 166.67 (4) S10A—Bi1—Br1^i^ 108.0 (3)

Br3—Bi1—Br2 91.31 (4) S10A—Bi1—Br1 78.8 (3)

Br3—Bi1—S30 99.55 (13) S10A—Bi1—S30A 170.9 (4)

Br3—Bi1—S30A 85.8 (3) Bi1^i^—Br1—Bi1 86.50 (3)

Br3—Bi1—S10 95.85 (14)

Symmetry code(s): (i) −*x*+1, *y*, −*z*+1/2.

(β-phase)

Bi2—Br2^i^ 2.9746 (10) Bi1—Br2^i^ 3.0173 (10)

Bi2—Br2 2.9746 (10) Bi1—Br2 3.0173 (10)

Bi2—Br3 2.7269 (10) Bi1—Br1^i^ 2.7065 (10)

Bi2—Br3^i^ 2.7268 (10) Bi1—Br1 2.7065 (10)

Bi2—S30^i^ 2.818 (6) Bi1—S10^i^ 2.815 (4)

Bi2—S30 2.818 (6) Bi1—S10 2.815 (4)

Bi2—S30A 2.94 (3) Bi1—S10A^i^ 2.914 (15)

Bi2—S30A^i^ 2.94 (3) Bi1—S10A 2.914 (15)

Br2^i^—Bi2—Br2 93.32 (4) Br1^i^—Bi1—Br2 167.60 (3)

Br3—Bi2—Br2^i^ 167.24 (3) Br1^i^—Bi1—Br2^i^ 89.15 (3)

Br3^i^—Bi2—Br2 167.24 (3) Br1—Bi1—Br2 89.15 (3)

Br3—Bi2—Br2 89.04 (3) Br1—Bi1—Br2^i^ 167.60 (3)

Br3^i^—Bi2—Br2^i^ 89.04 (3) Br1^i^—Bi1—Br1 92.75 (5)

Br3^i^—Bi2—Br3 91.41 (5) Br1—Bi1—S10^i^ 95.99 (10)

Br3—Bi2—S30^i^ 99.21 (12) Br1—Bi1—S10 95.41 (10)

Br3—Bi2—S30 95.36 (14) Br1^i^—Bi1—S10 95.99 (10)

Br3^i^—Bi2—S30^i^ 95.36 (14) Br1^i^—Bi1—S10^i^ 95.41 (10)

Br3^i^—Bi2—S30 99.21 (12) Br1^i^—Bi1—S10A 83.2 (3)

Br3^i^—Bi2—S30A 87.9 (5) Br1^i^—Bi1—S10A^i^ 84.9 (3)

Br3—Bi2—S30A 88.5 (7) Br1—Bi1—S10A^i^ 83.2 (3)

Br3—Bi2—S30A^i^ 87.9 (5) Br1—Bi1—S10A 84.9 (3)

Br3^i^—Bi2—S30A^i^ 88.5 (7) S10—Bi1—Br2 96.03 (10)

S30^i^—Bi2—Br2 71.99 (14) S10^i^—Bi1—Br2 72.21 (10)

S30—Bi2—Br2 93.44 (12) S10—Bi1—Br2^i^ 72.21 (10)

S30—Bi2—Br2^i^ 71.99 (14) S10^i^—Bi1—Br2^i^ 96.03 (10)

S30^i^—Bi2—Br2^i^ 93.44 (12) S10^i^—Bi1—S10 163.5 (2)

S30^i^—Bi2—S30 159.1 (3) S10A^i^—Bi1—Br2 83.1 (4)

S30A^i^—Bi2—Br2 78.8 (7) S10A—Bi1—Br2^i^ 83.1 (4)

S30A—Bi2—Br2^i^ 78.8 (7) S10A^i^—Bi1—Br2^i^ 109.2 (3)

S30A—Bi2—Br2 104.9 (5) S10A—Bi1—Br2 109.2 (3)

S30A^i^—Bi2—Br2^i^ 104.9 (5) S10A—Bi1—S10A^i^ 162.8 (8)

S30A^i^—Bi2—S30A 174.8 (13) Bi2—Br2—Bi1 87.54 (2)

Br2^i^—Bi1—Br2 91.61 (4)

Symmetry code(s): (i) −*x*, *y*, −*z*+1/2.

(γ-phase)

Bi1—Br1 3.0018 (8) Bi1—S10 2.841 (12)

Bi1—Br1^i^ 3.0018 (8) Bi1—S10A^i^ 2.84 (4)

Bi1—Br2^i^ 2.7207 (10) Bi1—S10A 2.84 (4)

Bi1—Br2 2.7207 (10) Br1—Bi1^ii^ 3.0018 (8)

Bi1—S10^i^ 2.842 (12)

Br1—Bi1—Br1^i^ 91.70 (3) Br2^i^—Bi1—S10A 89.5 (8)

Br2^i^—Bi1—Br1^i^ 89.49 (3) S10—Bi1—Br1^i^ 94.21 (16)

Br2—Bi1—Br1 89.49 (3) S10^i^—Bi1—Br1 94.21 (16)

Br2—Bi1—Br1^i^ 167.63 (2) S10^i^—Bi1—Br1^i^ 72.4 (2)

Br2^i^—Bi1—Br1 167.63 (2) S10—Bi1—Br1 72.4 (2)

Br2—Bi1—Br2^i^ 91.99 (5) S10—Bi1—S10^i^ 161.1 (4)

Br2—Bi1—S10^i^ 95.2 (2) S10A—Bi1—Br1^i^ 104.4 (5)

Br2—Bi1—S10 97.89 (16) S10A^i^—Bi1—Br1 104.4 (5)

Br2^i^—Bi1—S10 95.2 (2) S10A^i^—Bi1—Br1^i^ 78.2 (8)

Br2^i^—Bi1—S10^i^ 97.89 (16) S10A—Bi1—Br1 78.2 (8)

Br2—Bi1—S10A 87.9 (5) S10A^i^—Bi1—S10A 176.3 (12)

Br2^i^—Bi1—S10A^i^ 87.9 (5) Bi1^ii^—Br1—Bi1 88.30 (3)

Br2—Bi1—S10A^i^ 89.5 (8)

Symmetry code(s): (i) −*x*+1, *y*, −*z*+1/2; (ii) −*x*+1, −*y*+3/2, *z*.

**Table S2**. Geometry of the intermolecular contacts in **1** and **2** (in italics) in **γ** phase (Å, º)

| D-H···A | D-H | D···A | H···A | < D-H···A | Symmetry codes for acceptor |
| --- | --- | --- | --- | --- | --- |
| N-H···Cl | 0.86 | 3.68(1) | 3.02 | 135 | ½+x,1-y,1/2-z |
| N-H···Br | 0.86 | 3.723(7) | 3.01 | 142 | ½+x,1-y,1/2-z |
| C-H···Cl | 0.97 | 3.78(2) | 2.86 | 157 | 3/2-x,1-y,z |
| C-H···Br | 0.97 | 3.86(1) | 2.96 | 155 | 3/2-x,1-y,z |
| C-H···S (1) | 0.93 | 3.85(1) | 3.07 | 143 | 3/2-x,y,1/2+z |
| C-H···S (2) | 0.93 | 3.97(1) | 3.19 | 142.5 | 3/2-x,y,1/2+z |

**Table S3**. Unit cell parameters in different temperatures

**Complex 1**

| **T[K]** | **a [Å]** | | **b[Å]** | | **c[Å]** | | **α[o]** | | **β [o]** | | **γ [o]** | | **V [Å3]** | |
| --- | --- | --- | --- | --- | --- | --- | --- | --- | --- | --- | --- | --- | --- | --- |
| **100** | 14,79 | 16,49 | | 23,30 | | 90,00 | | 94,46 | | 90,00 | | 5666,20 | |  |
| **105** | 14,79 | 16,49 | | 23,29 | | 90,00 | | 94,42 | | 90,00 | | 5664,05 | |  |
| **110** | 14,80 | 16,49 | | 23,29 | | 90,00 | | 94,41 | | 90,00 | | 5667,91 | |  |
| **115** | 14,82 | 16,51 | | 23,30 | | 90,00 | | 94,34 | | 90,00 | | 5684,80 | |  |
| **120** | 14,82 | 16,52 | | 23,30 | | 90,00 | | 94,29 | | 90,00 | | 5690,95 | |  |
| **125** | 14,82 | 16,51 | | 23,30 | | 90,00 | | 94,33 | | 90,00 | | 5685,66 | |  |
| **130** | 14,83 | 16,51 | | 23,30 | | 90,00 | | 94,26 | | 90,00 | | 5691,15 | |  |
| **135** | 14,84 | 16,53 | | 23,32 | | 90,00 | | 94,07 | | 90,00 | | 5703,34 | |  |
| **140** | 14,85 | 16,55 | | 23,30 | | 90,00 | | 93,95 | | 90,00 | | 5711,50 | |  |
| **145** | 14,86 | 16,54 | | 23,31 | | 90,00 | | 93,91 | | 90,00 | | 5714,49 | |  |
| **150** | 14,86 | 16,55 | | 23,30 | | 90,00 | | 93,77 | | 90,00 | | 5720,93 | |  |
| **155** | 14,87 | 16,54 | | 23,29 | | 90,00 | | 93,73 | | 90,00 | | 5714,99 | |  |
| **160** | 14,87 | 16,54 | | 23,29 | | 90,00 | | 93,63 | | 90,00 | | 5719,24 | |  |
| **165** | 14,88 | 16,55 | | 23,30 | | 90,00 | | 93,56 | | 90,00 | | 5726,99 | |  |
| **170** | 14,89 | 16,56 | | 23,30 | | 90,00 | | 93,46 | | 90,00 | | 5732,70 | |  |
| **175** | 14,90 | 16,56 | | 23,29 | | 90,00 | | 93,50 | | 90,00 | | 5737,69 | |  |
| **180** | 14,90 | 16,57 | | 23,30 | | 90,00 | | 93,50 | | 90,00 | | 5743,00 | |  |
| **185** | 14,91 | 16,58 | | 23,30 | | 90,00 | | 93,44 | | 90,00 | | 5747,00 | |  |
| **190** | 14,93 | 16,58 | | 23,31 | | 90,00 | | 93,30 | | 90,00 | | 5762,04 | |  |
| **195** | 14,94 | 16,59 | | 23,31 | | 90,00 | | 93,33 | | 90,00 | | 5768,76 | |  |
| **200** | 14,95 | 16,59 | | 23,32 | | 90,00 | | 93,31 | | 90,00 | | 5774,14 | |  |
| **205** | 14,97 | 16,61 | | 23,33 | | 90,00 | | 93,20 | | 90,00 | | 5788,89 | |  |
| **210** | 14,98 | 16,62 | | 23,33 | | 90,00 | | 93,16 | | 90,00 | | 5796,33 | |  |
| **215** | 14,98 | 16,62 | | 23,33 | | 90,00 | | 93,12 | | 90,00 | | 5798,29 | |  |
| **220** | 14,99 | 16,62 | | 23,33 | | 90,00 | | 92,97 | | 90,00 | | 5806,69 | |  |
| **225** | 15,01 | 16,63 | | 23,33 | | 90,00 | | 92,76 | | 90,00 | | 5816,06 | |  |
| **230** | 15,02 | 16,63 | | 23,33 | | 90,00 | | 92,51 | | 90,00 | | 5821,86 | |  |
| **235** | 15,05 | 16,65 | | 23,31 | | 90,00 | | 90,00 | | 90,00 | | 5840,00 | |  |
| **240** | 15,06 | 16,66 | | 23,32 | | 90,00 | | 90,00 | | 90,00 | | 5849,21 | |  |
| **245** | 15,07 | 16,66 | | 23,32 | | 90,00 | | 90,00 | | 90,00 | | 5855,01 | |  |
| **250** | 15,08 | 16,66 | | 23,32 | | 90,00 | | 90,00 | | 90,00 | | 5859,92 | |  |
| **255** | 15,09 | 16,66 | | 23,32 | | 90,00 | | 90,00 | | 90,00 | | 5864,92 | |  |
| **260** | 15,10 | 16,67 | | 23,33 | | 90,00 | | 90,00 | | 90,00 | | 5874,27 | |  |
| **265** | 15,12 | 16,68 | | 23,33 | | 90,00 | | 90,00 | | 90,00 | | 5883,85 | |  |
| **270** | 15,13 | 16,68 | | 23,34 | | 90,00 | | 90,00 | | 90,00 | | 5892,18 | |  |
| **275** | 15,15 | 16,68 | | 23,34 | | 90,00 | | 90,00 | | 90,00 | | 5897,62 | |  |
| **280** | 15,16 | 16,69 | | 23,34 | | 90,00 | | 90,00 | | 90,00 | | 5908,24 | |  |
| **285** | 15,18 | 16,70 | | 23,35 | | 90,00 | | 90,00 | | 90,00 | | 5921,10 | |  |
| **290** | 15,20 | 16,71 | | 23,36 | | 90,00 | | 90,00 | | 90,00 | | 5934,69 | |  |
| **295** | 15,22 | 16,72 | | 23,38 | | 90,00 | | 90,00 | | 90,00 | | 5950,28 | |  |
| **300** | 15,24 | 16,73 | | 23,38 | | 90,00 | | 90,00 | | 90,00 | | 5961,74 | |  |

**Complex 2**

| **T[K]** | **a [Å]** | **b[Å]** | **c[Å]** | **α[^o^]** | **β [^o^]** | **γ [^o^]** | **V [Å^3^]** |
| --- | --- | --- | --- | --- | --- | --- | --- |
| **100** | 15,15 | 16,95 | 23,47 | 89,99 | 92,88 | 89,95 | 6019,38 |
| **105** | 15,15 | 16,95 | 23,45 | 90,00 | 92,98 | 90,02 | 6010,38 |
| **110** | 15,19 | 17,00 | 23,52 | 89,99 | 92,94 | 89,94 | 6065,45 |
| **115** | 15,16 | 16,96 | 23,46 | 89,99 | 92,93 | 89,95 | 6023,90 |
| **120** | 15,20 | 17,00 | 23,51 | 90,01 | 92,90 | 90,05 | 6062,78 |
| **125** | 15,22 | 17,01 | 23,54 | 90,02 | 92,83 | 90,06 | 6086,14 |
| **130** | 15,23 | 17,02 | 23,53 | 90,02 | 92,85 | 90,07 | 6089,53 |
| **135** | 15,19 | 16,97 | 23,46 | 89,99 | 92,81 | 89,95 | 6038,53 |
| **140** | 15,20 | 16,98 | 23,46 | 89,97 | 92,76 | 89,94 | 6045,69 |
| **145** | 15,22 | 17,00 | 23,51 | 89,98 | 92,69 | 89,93 | 6076,38 |
| **150** | 15,22 | 17,00 | 23,50 | 89,97 | 92,63 | 89,93 | 6076,49 |
| **155** | 15,21 | 16,98 | 23,48 | 90,02 | 92,64 | 90,06 | 6058,09 |
| **160** | 15,23 | 16,98 | 23,47 | 89,97 | 92,58 | 89,90 | 6064,23 |
| **165** | 15,27 | 16,95 | 23,48 | 90,01 | 92,50 | 90,01 | 6067,13 |
| **170** | 15,26 | 16,93 | 23,45 | 89,99 | 92,49 | 89,94 | 6053,46 |
| **175** | 15,33 | 17,01 | 23,53 | 90,05 | 92,42 | 90,08 | 6129,31 |
| **180** | 15,32 | 17,07 | 23,46 | 89,91 | 89,98 | 89,88 | 6133,04 |
| **185** | 15,31 | 17,10 | 23,45 | 89,94 | 89,97 | 89,87 | 6136,28 |
| **190** | 15,33 | 17,12 | 23,47 | 89,94 | 89,98 | 89,89 | 6158,88 |
| **195** | 15,32 | 17,11 | 23,44 | 89,97 | 89,95 | 89,90 | 6143,36 |
| **200** | 15,35 | 17,13 | 23,48 | 90,07 | 90,03 | 90,08 | 6173,54 |
| **205** | 15,34 | 17,13 | 23,45 | 89,97 | 89,98 | 89,89 | 6163,14 |
| **210** | 15,38 | 17,16 | 23,50 | 90,07 | 90,03 | 90,11 | 6200,25 |
| **215** | 15,38 | 17,15 | 23,47 | 89,97 | 90,00 | 89,89 | 6189,61 |
| **220** | 15,38 | 17,15 | 23,46 | 90,02 | 90,00 | 90,10 | 6188,39 |
| **225** | 15,39 | 17,15 | 23,48 | 90,02 | 90,01 | 90,10 | 6195,78 |
| **230** | 15,41 | 17,17 | 23,48 | 89,96 | 89,99 | 89,87 | 6213,88 |
| **235** | 15,43 | 17,19 | 23,50 | 89,97 | 89,98 | 89,88 | 6232,64 |
| **240** | 15,43 | 17,18 | 23,50 | 89,98 | 89,98 | 89,88 | 6229,66 |
| **245** | 15,43 | 17,18 | 23,49 | 90,00 | 90,03 | 90,12 | 6230,05 |
| **250** | 15,45 | 17,19 | 23,51 | 89,99 | 89,97 | 89,89 | 6245,61 |
| **255** | 15,46 | 17,19 | 23,51 | 90,03 | 90,01 | 90,11 | 6247,04 |
| **260** | 15,46 | 17,18 | 23,50 | 90,05 | 90,02 | 90,12 | 6244,73 |
| **265** | 15,48 | 17,19 | 23,51 | 89,95 | 90,00 | 89,84 | 6259,14 |
| **270** | 15,48 | 17,19 | 23,50 | 90,06 | 90,00 | 90,14 | 6254,42 |
| **275** | 15,49 | 17,20 | 23,52 | 90,02 | 90,01 | 90,11 | 6266,03 |
| **280** | 15,51 | 17,20 | 23,52 | 90,05 | 90,01 | 90,15 | 6272,01 |
| **285** | 15,52 | 17,21 | 23,52 | 90,02 | 90,04 | 90,13 | 6282,00 |
| **290** | 15,54 | 17,21 | 23,53 | 89,95 | 90,00 | 89,85 | 6293,53 |
| **295** | 15,54 | 17,22 | 23,54 | 89,99 | 89,98 | 89,89 | 6298,98 |
| **300** | 15,55 | 17,22 | 23,58 | 90,00 | 90,09 | 90,08 | 6316,49 |
| **305** | 15,55 | 17,22 | 23,57 | 90,09 | 90,03 | 90,05 | 6308,95 |
| **310** | 15,55 | 17,20 | 23,56 | 90,13 | 90,03 | 90,04 | 6302,79 |
| **295** | 15,54 | 17,22 | 23,54 | 89,99 | 89,98 | 89,89 | 6298,98 |
| **300** | 15,55 | 17,22 | 23,58 | 90,00 | 90,09 | 90,08 | 6316,49 |
| **305** | 15,55 | 17,22 | 23,57 | 90,09 | 90,03 | 90,05 | 6308,95 |
| **310** | 15,55 | 17,20 | 23,56 | 90,13 | 90,03 | 90,04 | 6302,79 |

**Table S4.** Isostructurality index calculated for analogues phases

| Phases |  | Isostructural index % |
| --- | --- | --- |
| γ-γ |  | 99,30 |
| δ-γ |  | 99,29 |
| β-β |  | 98,77 |
| α-α |  | 97,69 / 97,38 |

**Table S5**. The systematic analysis of reflection extinction in different temperatures also indicates some structural and symmetry changes.

|  |  | Hkl | R.condition/temperature | 100 | 190 | 220 | 230 | 240 | 298 | 303 | 333 |
| --- | --- | --- | --- | --- | --- | --- | --- | --- | --- | --- | --- |
| C1 | b\|x | 0kl | k=2n+1 | 2.6 | 2.7 | 2.0 | 2.4 | 1.9 | 1.1 | 0.5 | 0.5 |
|  | c\|x | 0kl | l=2n+1 | 3.2 | 3.1 | 2.1 | 2.6 | 0.7 | 0.6 | 1.1 | 0.5 |
|  | n\|x | 0kl | k+l=2n+1 | 3.1 | 3.4 | 2.4 | 3.0 | 2.0 | 1.1 | 1.1 | 0.5 |
|  | a\|y | h0l | h=2n+1 | 2.1 | 2.5 | 1.8 | 2.0 | 3.1 | 1.6 | 1.5 | 0.8 |
|  | c\|y | h0l | l=2n+1 | 4.5 | 3.5 | 2.1 | 2.4 | 7.5 | 1.5 | 2.0 | 0.9 |
|  | n\|y | h0l | h+l=2n+1 | 5.8 | 4.5 | 3.0 | 3.4 | 7.7 | 2.7 | 3.1 | 0.5 |
|  | a\|z | hk0 | h=2n+1 | 3.6 | 4.6 | 4.0 | 4.6 | 7.5 | 2.8 | 2.9 | 0.4 |
|  | b\|z | hk0 | k=2n+1 | 3.3 | 3.4 | 3.0 | 3.6 | 6.8 | 1.4 | 1.8 | 1.0 |
|  | n\|z | hk0 | h+k=2n+1 | 3.0 | 2.9 | 2.3 | 2.6 | 3.4 | 2.1 | 1.9 | 1.0 |
|  | A | hkl | k+l=2n+1 | 4.3 | 4.0 | 2.9 | 3.4 | 6.5 | 2.5 | 2.8 | 1.5 |
|  | B | hkl | h+l=2n+1 | 4.4 | 4.3 | 3.1 | 3.7 | 6.3 | 2.8 | 3.1 | 1.5 |
|  | C | hkl | h+k=2n+1 | 2.6 | 2.5 | 1.9 | 2.2 | 2.8 | 1.6 | 1.4 | 0.4 |
|  | I | hkl | h+k+l=2n+1 | 6.5 | 6.5 | 4.8 | 5.5 | 7.5 | 5.9 | 6.2 | 3.7 |
|  | F | hkl | t all od | 3.8 | 3.6 | 2.6 | 3.1 | 5.2 | 2.3 | 2.4 | 1.1 |
|  |  |  |  |  |  |  |  |  |  |  |  |
|  |  | **hkl** | **R.condition/temperature** | **100** | **170** | **190** | **240** | **260** | **298** | **303** | **333** |
| 2 | b\|x | 0kl | k=2n+1 | 2.0 | 2.0 | 0.6 | 0.5 | 0.5 | 0.5 | 0.6 | 0.6 |
|  | c\|x | 0kl | l=2n+1 | 2.3 | 1.7 | 0.6 | 0.5 | 0.5 | 0.5 | 0.5 | 0.6 |
|  | n\|x | 0kl | k+l=2n+1 | 3.0 | 2.5 | 0.6 | 0.5 | 0.5 | 0.5 | 0.5 | 0.6 |
|  | a\|y | h0l | h=2n+1 | 1.2 | 1.3 | 0.4 | 0.4 | 0.4 | 0.4 | 0.4 | 0.4 |
|  | c\|y | h0l | l=2n+1 | 1.5 | 1.9 | 3.4 | 2.2 | 0.8 | 0.6 | 0.5 | 0.5 |
|  | n\|y | h0l | h+l=2n+1 | 2.1 | 2.1 | 3.4 | 2.3 | 0.8 | 0.6 | 0.5 | 0.5 |
|  | a\|z | hk0 | h=2n+1 | 2.5 | 2.3 | 3.0 | 2.0 | 0.7 | 0.6 | 0.6 | 0.6 |
|  | b\|z | hk0 | k=2n+1 | 2.7 | 2.6 | 3.1 | 2.1 | 0.8 | 0.6 | 0.5 | 0.6 |
|  | n\|z | hk0 | h+k=2n+1 | 1.0 | 1.2 | 0.5 | 0.4 | 0.4 | 0.5 | 0.5 | 0.5 |
|  | A | hkl | k+l=2n+1 | 2.3 | 2.2 | 2.7 | 2.1 | 1.3 | 1.2 | 1.3 | 1.3 |
|  | B | hkl | h+l=2n+1 | 2.4 | 2.2 | 2.7 | 2.1 | 1.3 | 1.2 | 1.3 | 1.3 |
|  | C | hkl | h+k=2n+1 | 1.4 | 1.3 | 0.4 | 0.4 | 0.4 | 0.4 | 0.4 | 0.4 |
|  | I | hkl | h+k+l=2n+1 | 3.0 | 2.8 | 2.9 | 2.6 | 2.3 | 2.2 | 2.4 | 2.3 |
|  | F | hkl | t all od | 2.0 | 1.9 | 1.9 | 1.5 | 1.0 | 1.0 | 1.0 | 1.0 |

**Table S6.** Kitaigorodskii packing index (%) calculated for more occupied position of disordered structures

|  | **α** | **β** | **γ** | **δ** |
| --- | --- | --- | --- | --- |
| **1** | 68,3 | 65,9 | 64,9 | 64,0 |
| **2** | 66,0 | 64,4 | 63,5 | - |

**Table S7**. Relevant crystallographic and refinement data

|  | Complex **1** | | | | Complex **2** | | | Solid solution |
| --- | --- | --- | --- | --- | --- | --- | --- | --- |
| Name | α phase | β phase | δ phase | γ phase | α phase | β phase | γ phase | Lt β phase |
| Chemical formula | C_44_H_60_Bi_2_Cl_6_N_12_S_4_ | | | | C_44_H_60_Bi_2_Br_6_N_12_S_4_ | | | C_44_H_60_Bi_2_Br_1.68_Cl_4.32_N_12_S_4_ |
| *M*_r_ | 1515.94 | | | | 1682.70 | | | 1708.90 |
| Crystal system,  space group | Monoclinic *P*2_1_/*c* | Monoclinic *C*2/*c* | Orthorhombic *Ccce* | Orthorhombic *Ccce* | Monoclinic *C*2/*c* | Monoclinic *C*2/*c* | Orthorhombic *Ccce* | Monoclinic *C*2/*c* |
| Temperature (K) | 100(1) | 250(1) | 298(1) | 333(1) | 100 (1) | 200 (1) | 293(2) | 100(1) |
| *a*, *b*, *c* (Å) | 14.8035 (4) 16.5246 (5) 23.2996 (7) | 6.6914(4) 15.0827(4) 23.3076 (5) | 15.2405(4)  16.7327(3) 23.3773 (4) | 16.7598 (3) 15.3581 (3) 23.3959 (4) | 15.1589 (7) 16.9552 (7) 23.4305 (8) | 17.0706 (3) 15.3215 (3) 23.3800 (4) | 17.1784 (4) 15.5345 (5) 23.4823 (6) | 17.0706 (3), 15.3215 (3), 23.3800 (4) |
| β (°) | 94.317 (2) | 90.056 (2) | 90 | 90 | 92.920 (4) | 90.2758 (14) | 90 | 90.2758 (14) |
| *V* (Å^3^) | 5683.4 (3) | 5867.7 (2) | 5961.5 (2) | 6022.10 (19) | 6014.3 (4) | 6114.90 (17) | 6266.4 (3) | 6114.90 (19) |
| *Z* | 4 | 4 | 4 | 4 | 4 | 4 | 4 | 4 |
| μ (mm^-1^) | 6.66 | 6.45 | 6.35 | 6.28 | 10.01 | 9.85 | 9.61 | 8.83 |
| Measured, independent and observed [*I* > 2s(*I*)] reflections | 12411, 12411, 9888 | 21650, 6360, 5585 | 12721, 3059, 2428 | 55089, 3658, 3039 | 6501, 6501, 4857 | 23200, 6286, 5161 | 14674, 3234, 2264 | 11447, 4782, 3803 |
| *R*_int_ | - | 0.027 | 0.026 | 0.031 | - | 0.037 | 0.039 | 0.049 |
| (sin θ/λ)_max_ (Å^-1^) | 0.641 | 0.668 | 0.639 | 0.672 | 0.640 | 0.641 | 0.639 | 0.665 |
| *R*[*F*^2^ > 2s(*F*^2^)],  *wR*(*F*^2^), *S* | 0.039, 0.106, 1.04 | 0.080,  0.201,  1.20 | 0.077 0.148, 1.49 | 0.079, 0.156, 1.46 | 0.053, 0.116, 1.00 | 0.047, 0.100, 1.19 | 0.050, 0.087, 1.31 | 0.043, 0.095, 1.03 |
| No. of reflections | 12411 | 6360 | 3059 | 3658 | 6501 | 6286 | 3234 | 4782 |
| No. of parameters | 623 | 374 | 198 | 207 | 360 | 392 | 207 | 394 |
| Δρ_max_, Δρ_min_  (e Å^‑3^) | 2.40, -1.73 | 4.17, −2.67 | 0.83, -1.11 | 1.53, -1.63 | 2.87, -2.44 | 1.26, -1.63 | 0.74, -0.64 | 2.14, -1.45 |


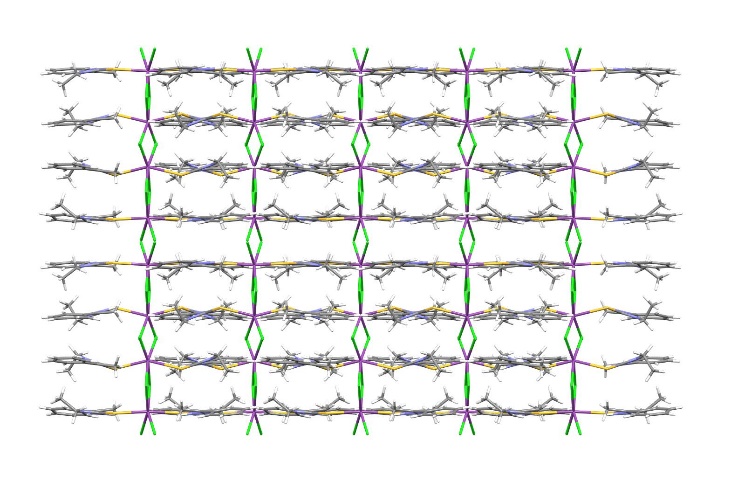

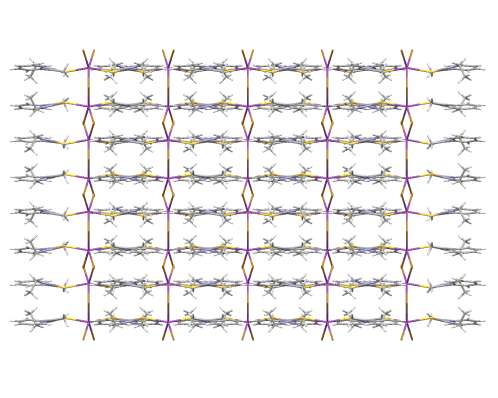


Room temperature


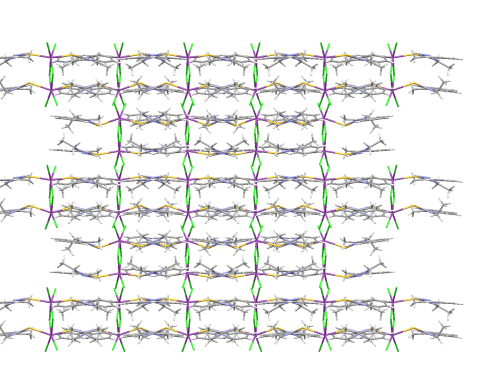

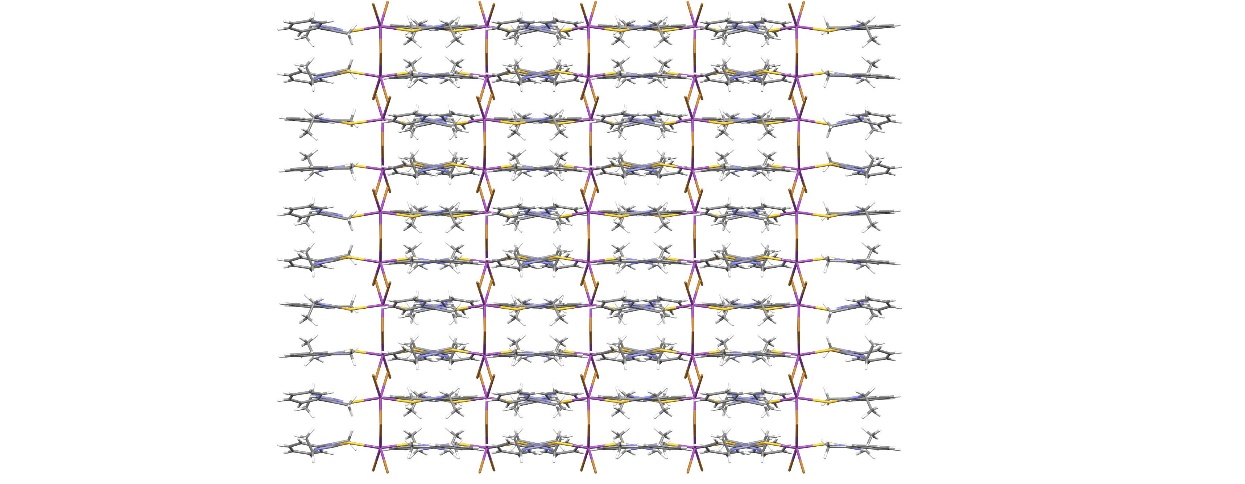


100K room temperature

**Figure S1.** The crystal packing along [001] direction for compounds **1** (left) and **2** (right) at 100K (lower panel) and at room temperature (upper panel)


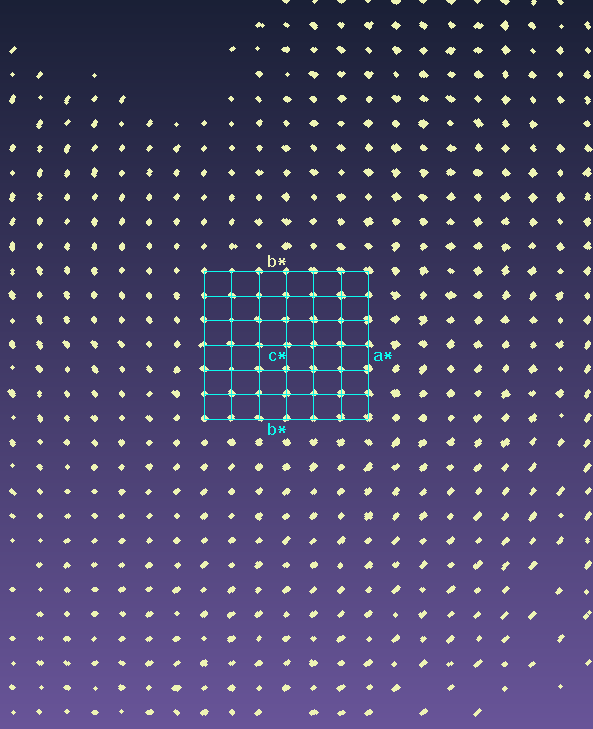

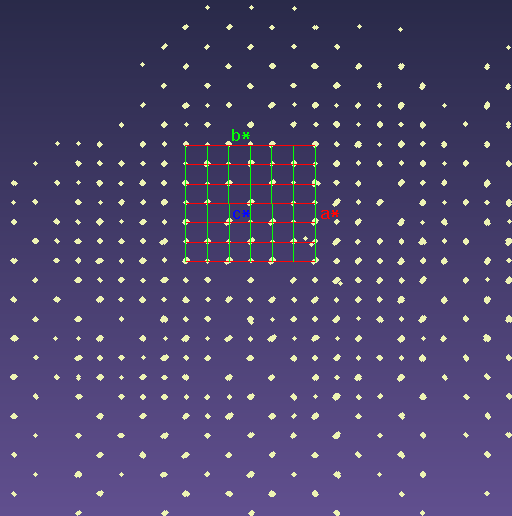

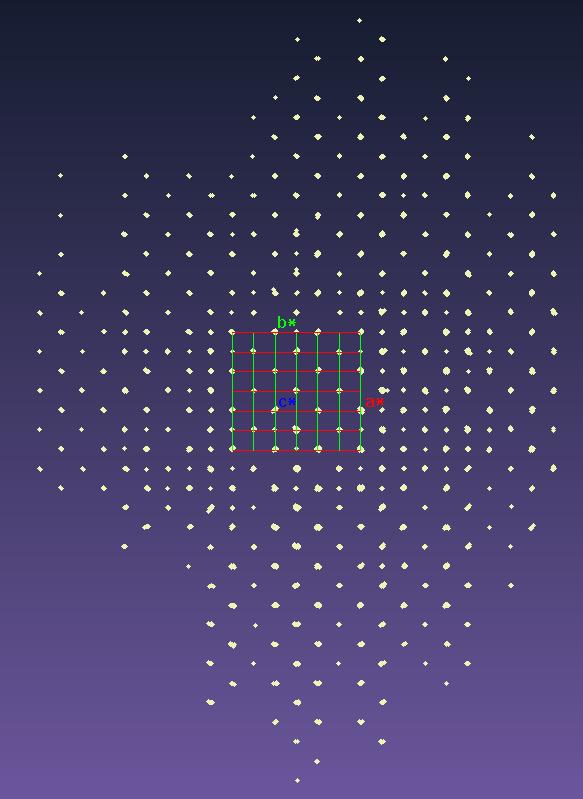

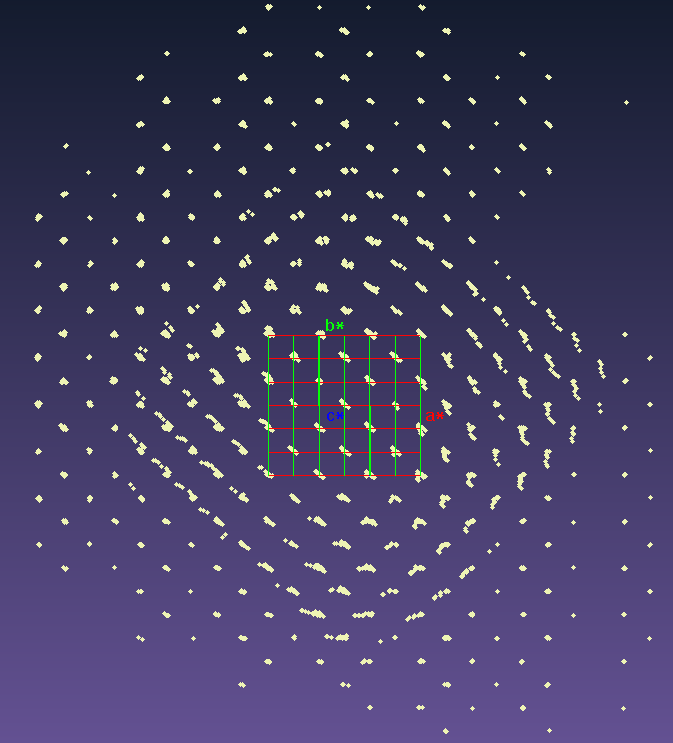


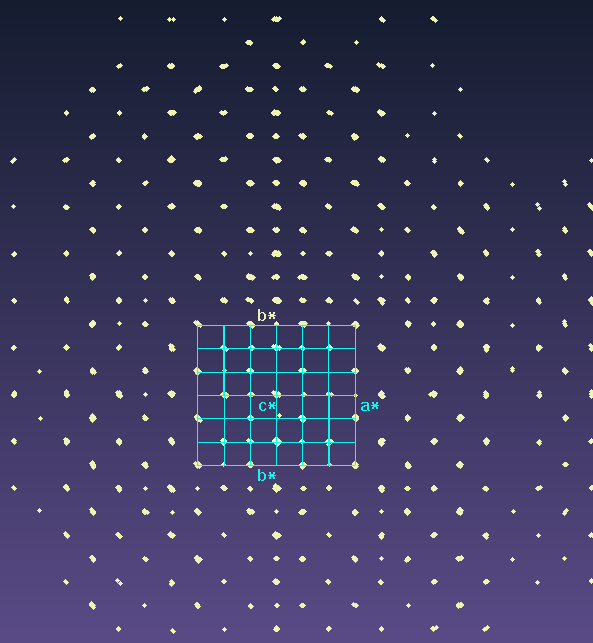

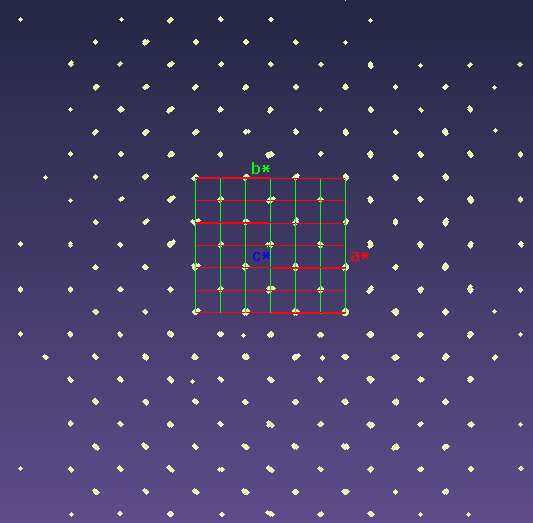

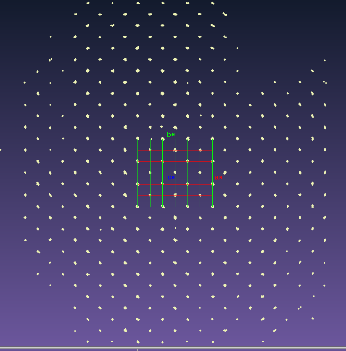

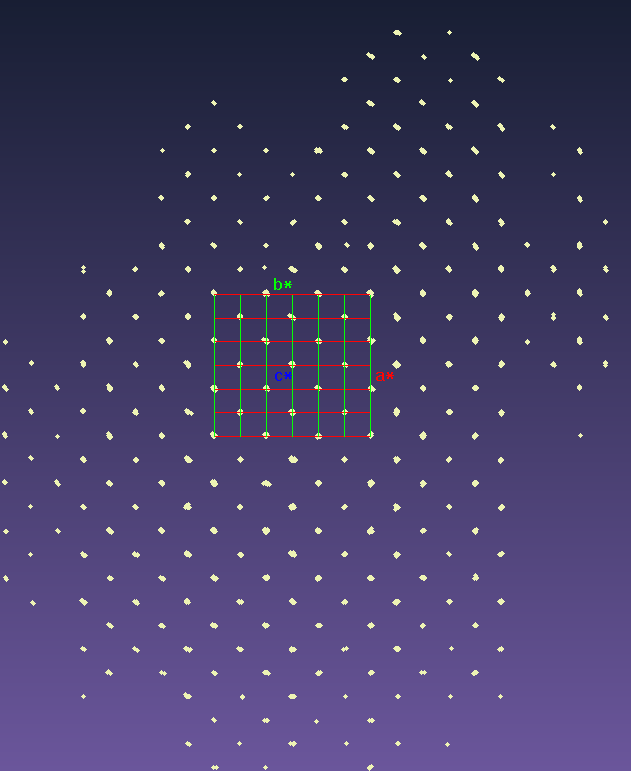


100 240 298 333

**Figure S2** Ewald representation along [001]for complexes **1** (top) and **2** (bottom) at different temperatures.

h1l h0l h0l


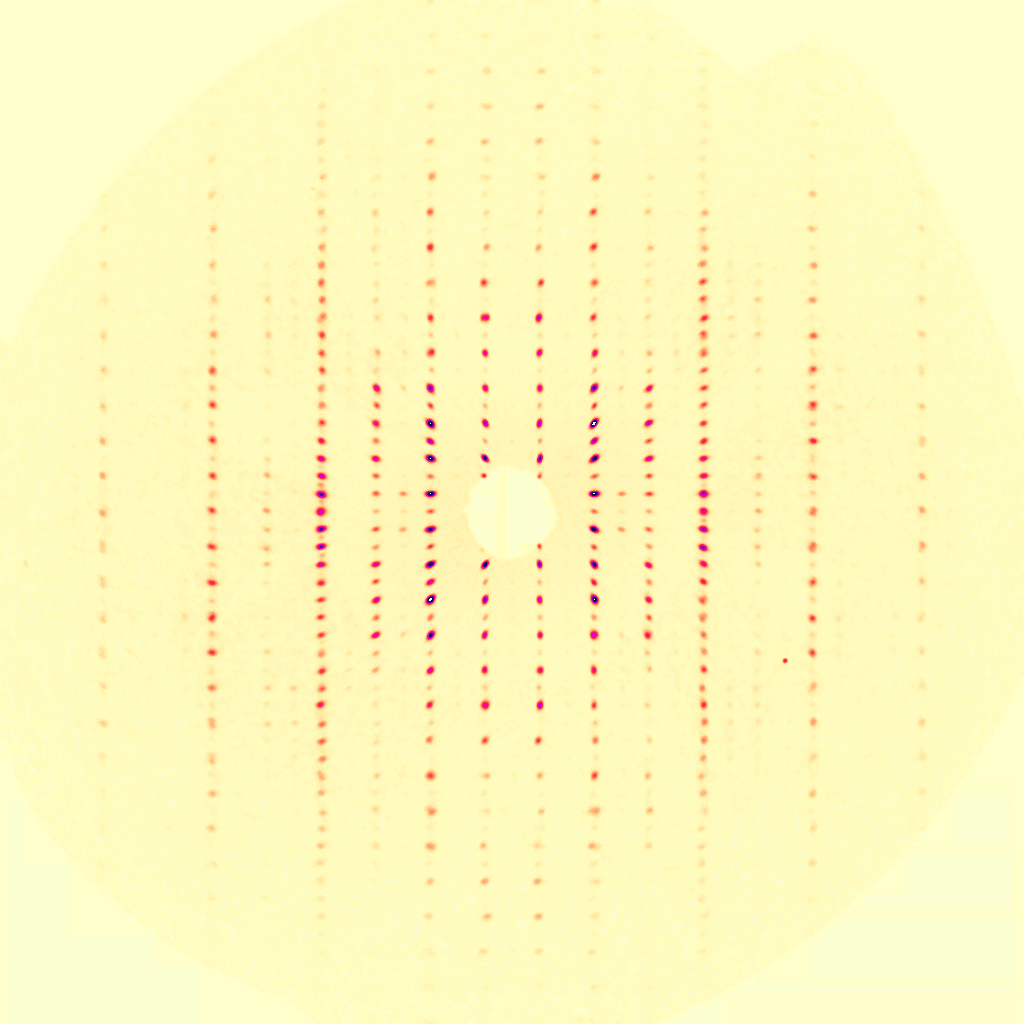

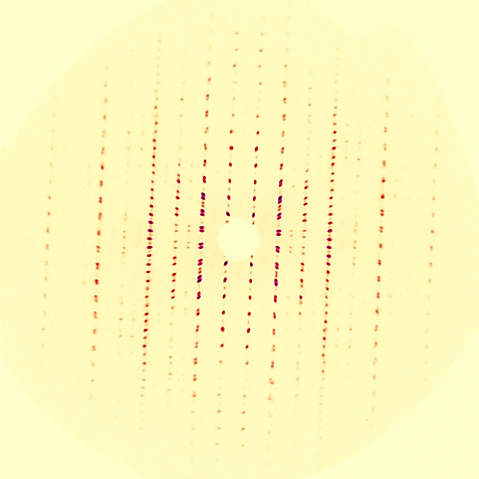

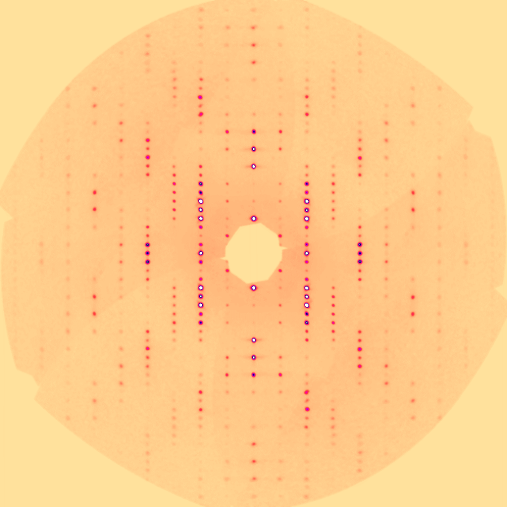

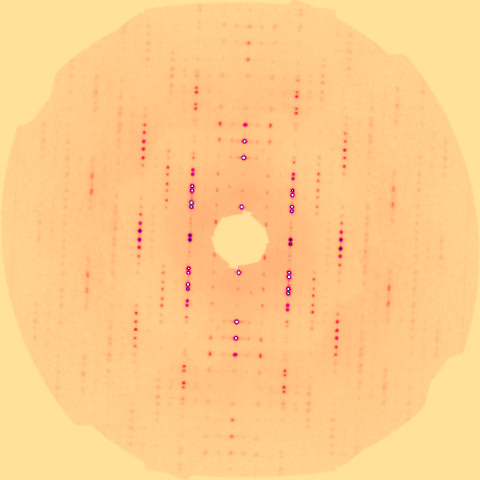


1. b)

235K 230K 190K 170K

**Figure S3.** The layers of crystal a) **1** and b) **2** in temperatures close to the phase transition from phase 2 to phase 1


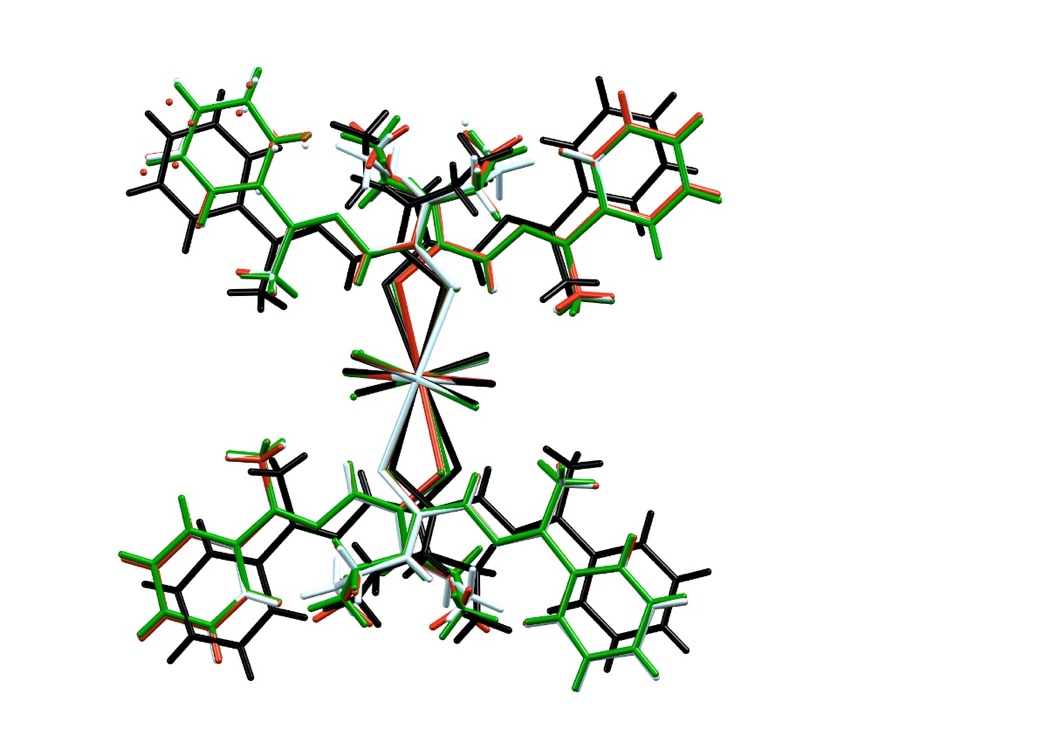

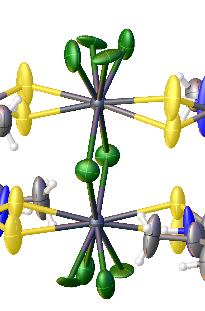

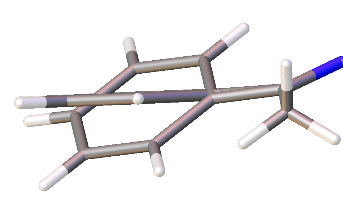

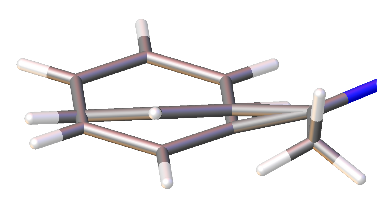


a) b) c)

**Figure S4**. A) The superposition of complex **1** molecules from different phases (**α**- black **β-**green **δ-**red **γ-** light blue. B) Disordered terminal chlorine atoms in **β** phase.
 c) Orientation of disordered phenyl rings in top-**γ-**phase and down **δ-phase.**


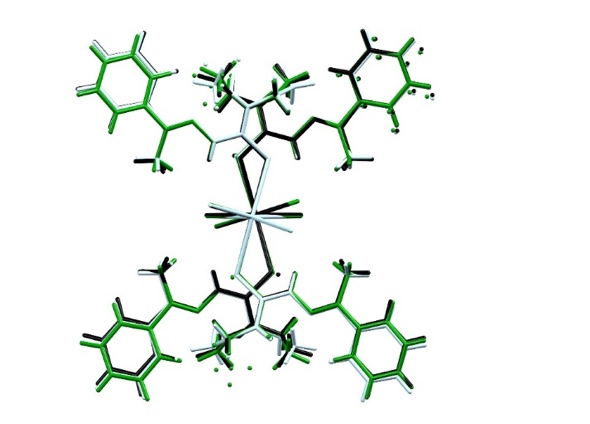

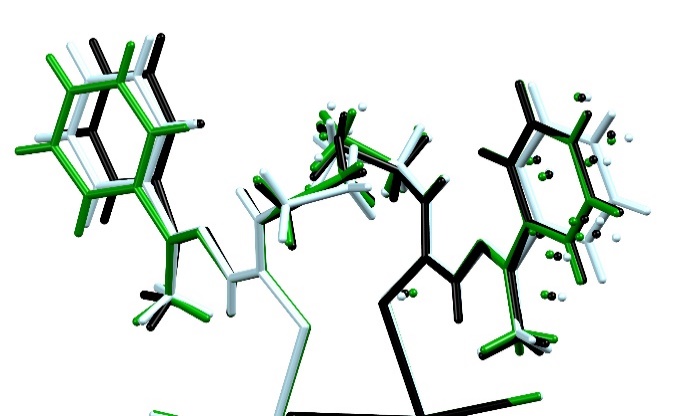

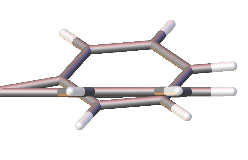

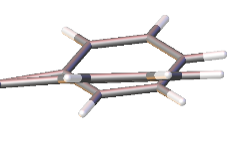

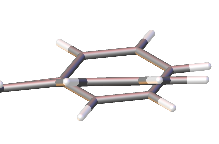


1. b) c) **α** d) **β** e) **γ**

Figure S5 a),b) The superposition of complex **2** molecules from different phases (**α**- black **β-**green **γ-** light blue from different views) Orientation of disordered phenyl rings in c) **α**, d) **β** e) **γ** phases.


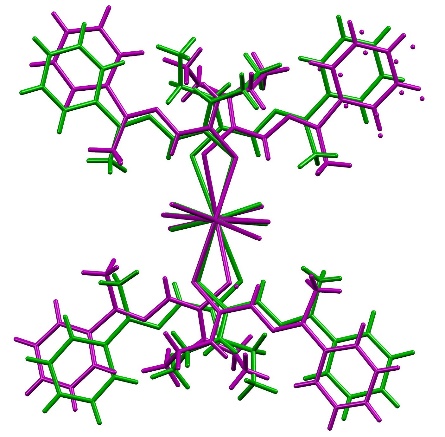

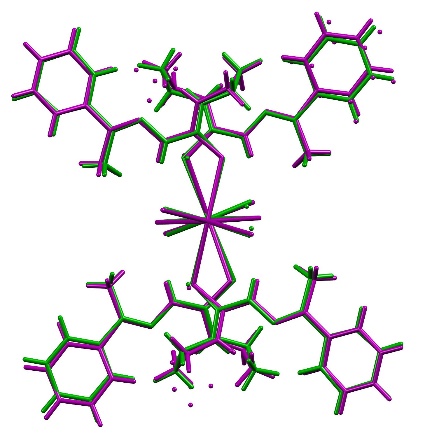

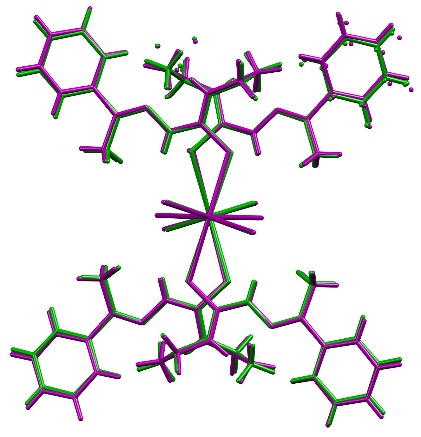


1. b) c)

**Figure S6**. Molecular overlay of the a) **α**, b) **β** c) **γ** phases of complexes **1** (green) and **2** (purple)

| Complex **1** | 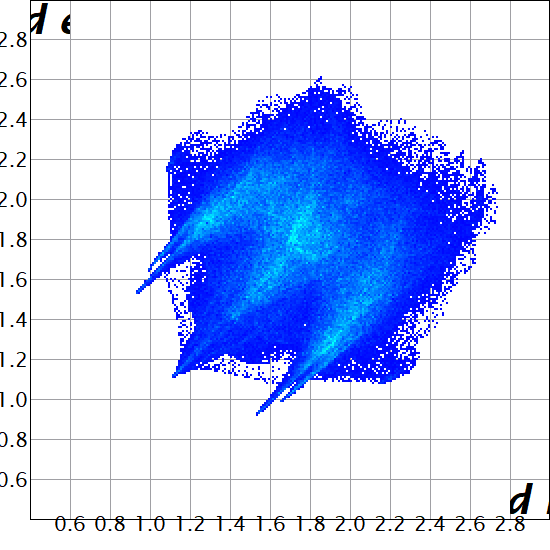 | 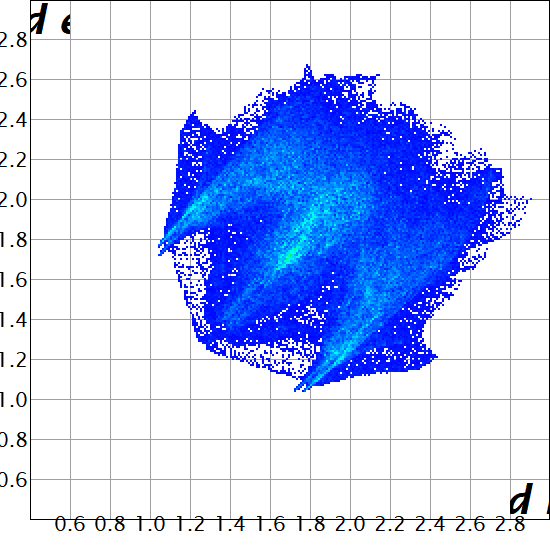 | 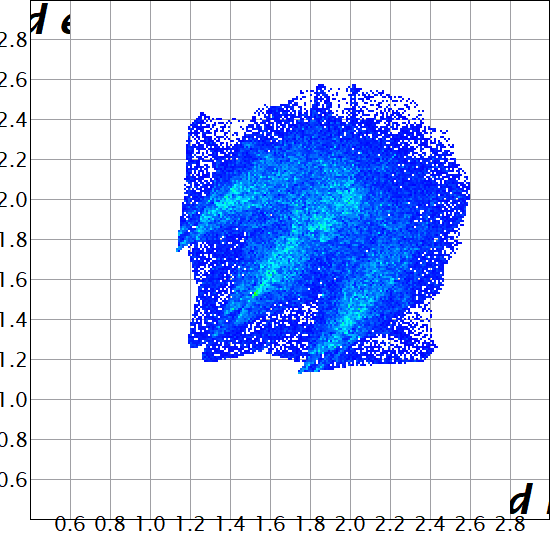 |
| --- | --- | --- | --- |
|  | **α** | **β** | **δ** |
| Complex **2** | 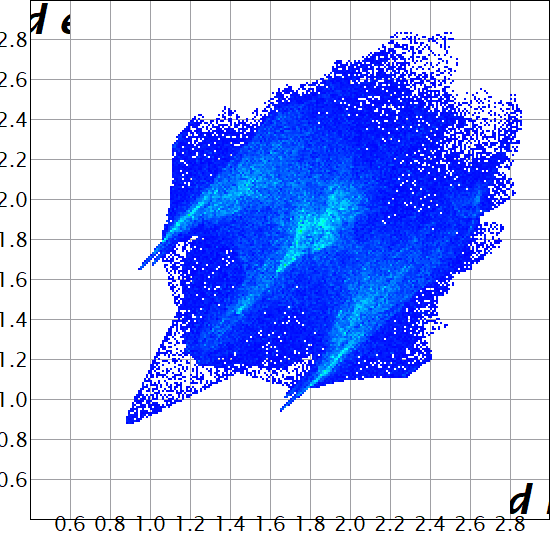 | 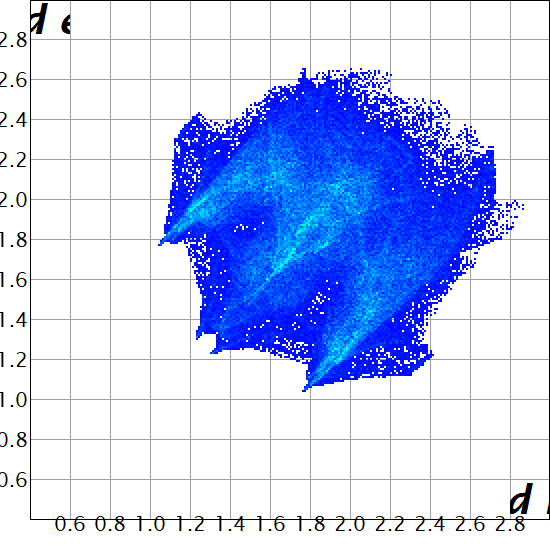 | 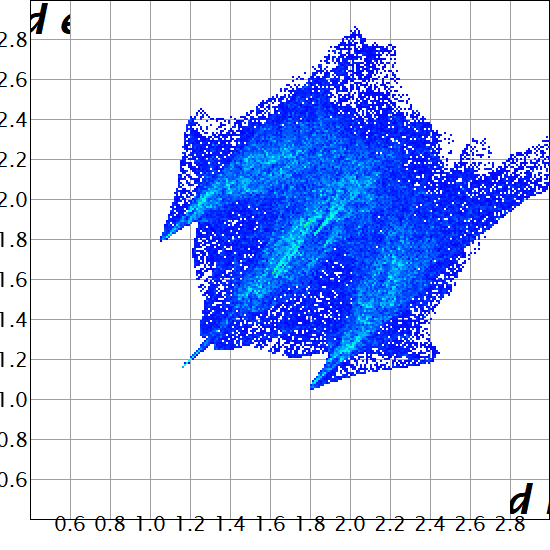 |
|  | **α** | **β** | **γ** |

**Figure S7.** Hirshfeld fingerplots of complex molecules in different phases.

**Fig. S8**. The unit cell similarity index and elongation vs. temperature [K]


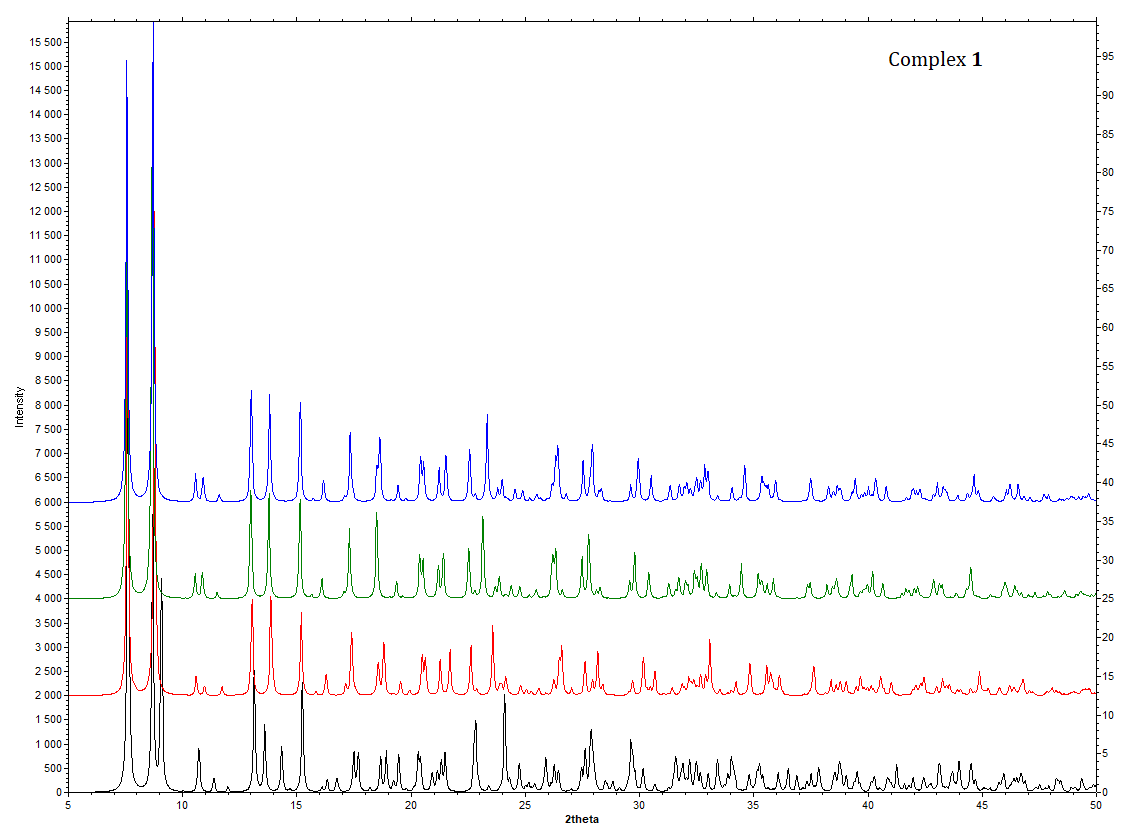


**Fig. S9.1.** Theoretically generated powder pattern for complex **1**: Black-α phase , green- β phase, red- γ phase, blue- δ phase


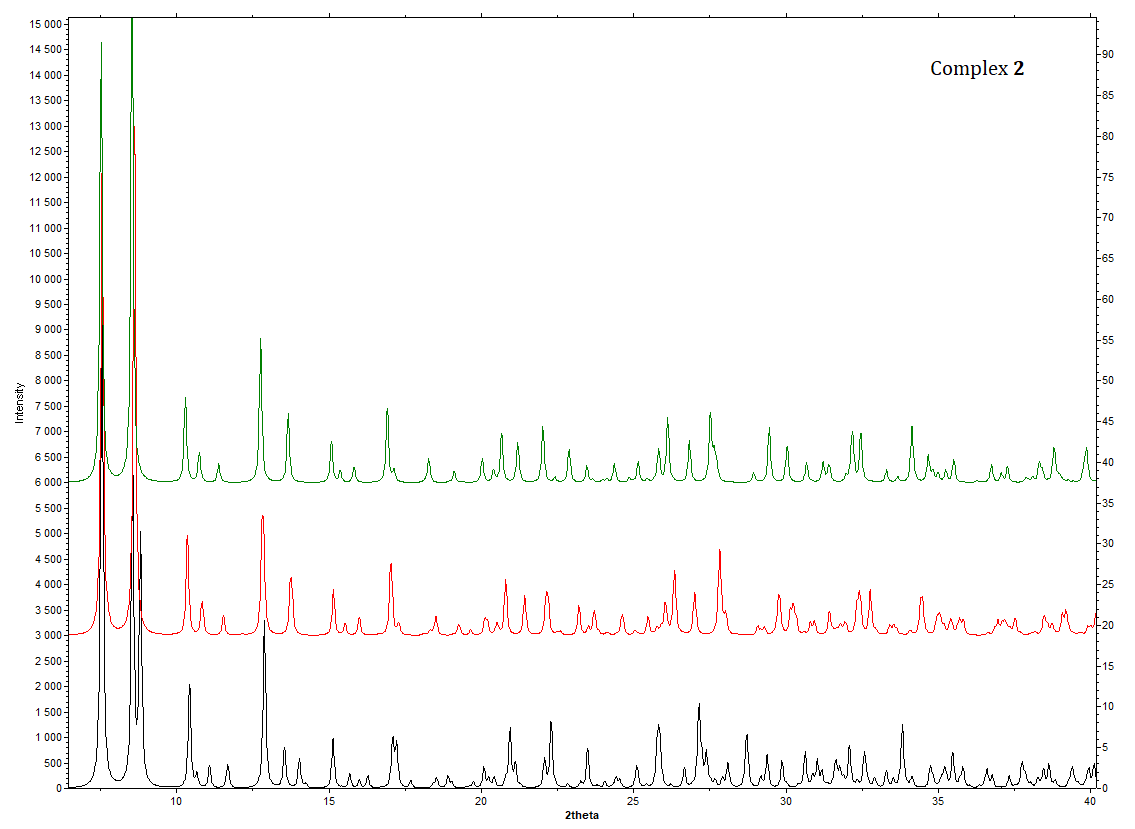


**Fig. S9.2.** Theoretically generated powder pattern for complex **2** : Black-α phase , green- β phase, red- γ phase.


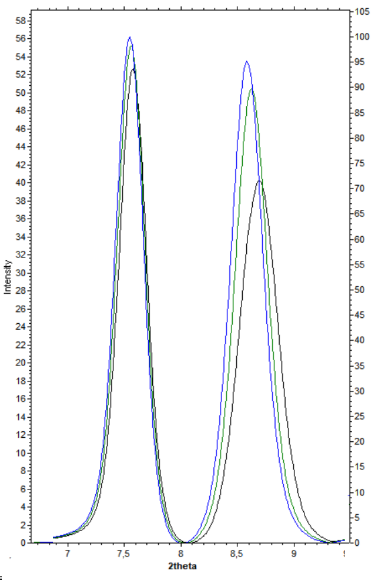

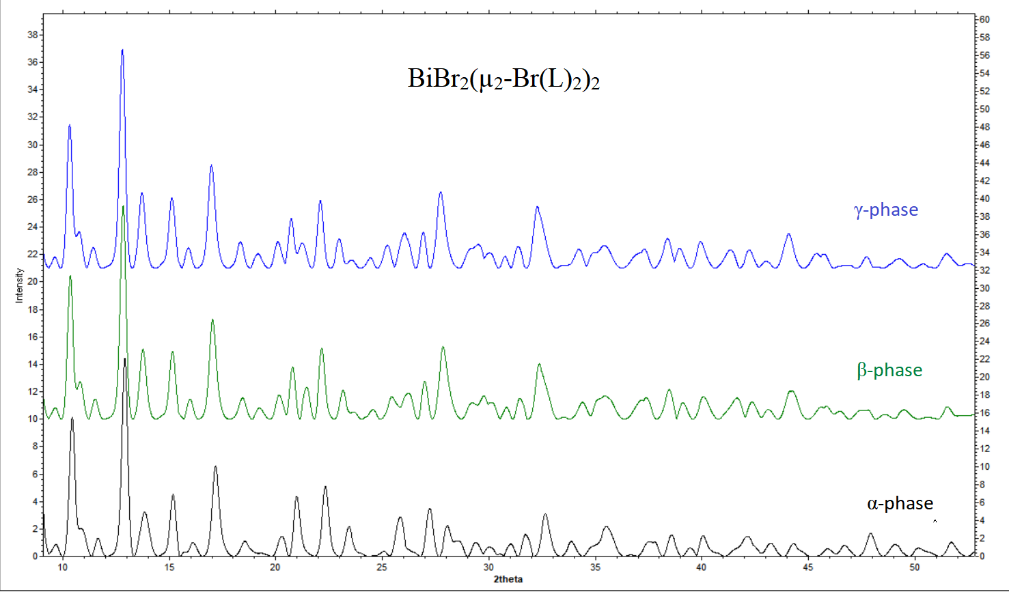


**Figure S10.** The powder X-Ray diffractogrmas collected for 130 K black, 240 K green, 290K blue one form complex **2**


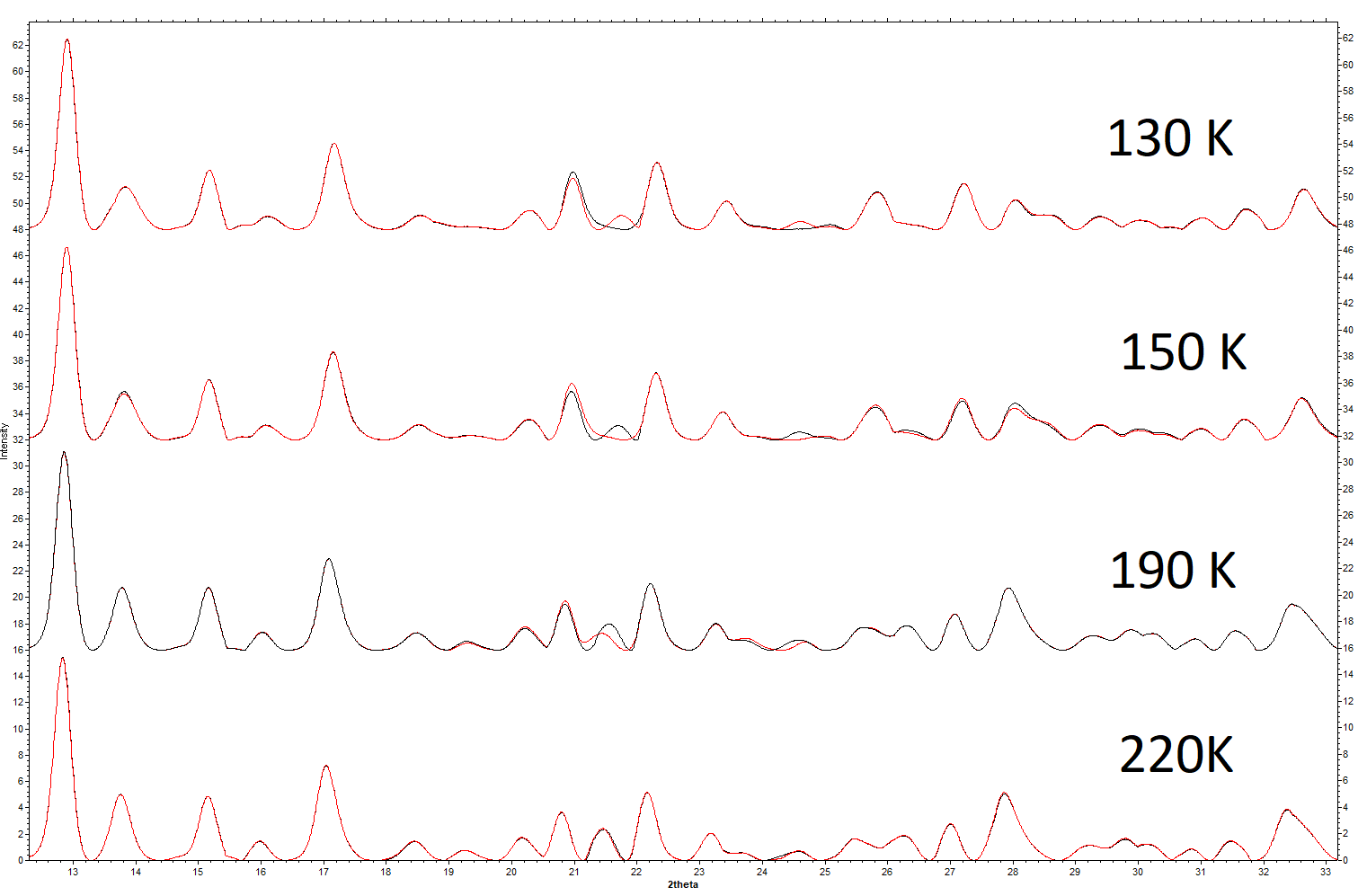


**Fig. S11.** Selected fragment of the temperature PXRD patterns for compound **2** at four temperatures: red – first measurement in the cycle, black -second measurement in the cycle.


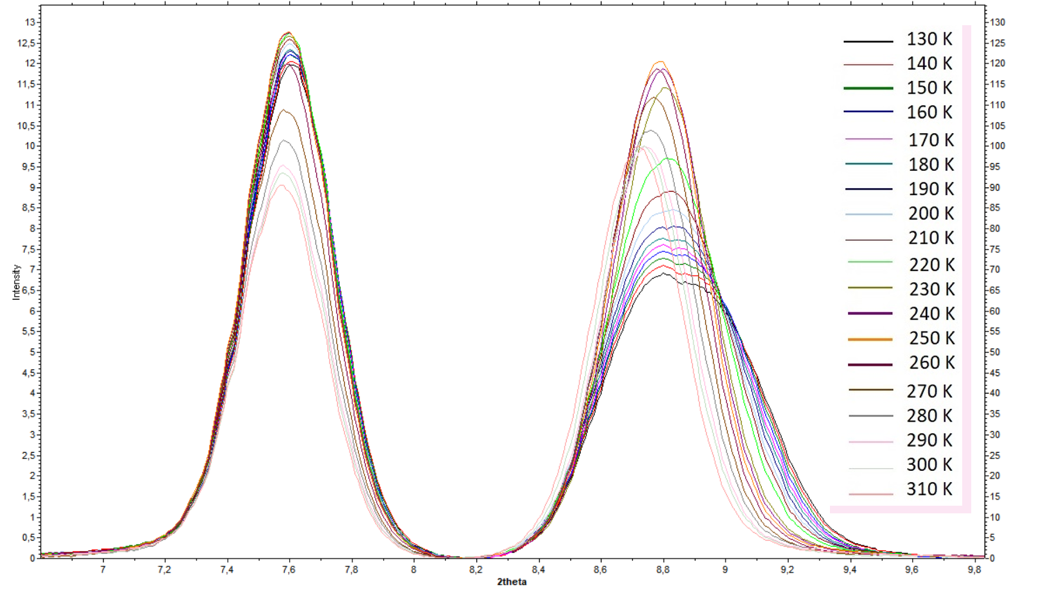

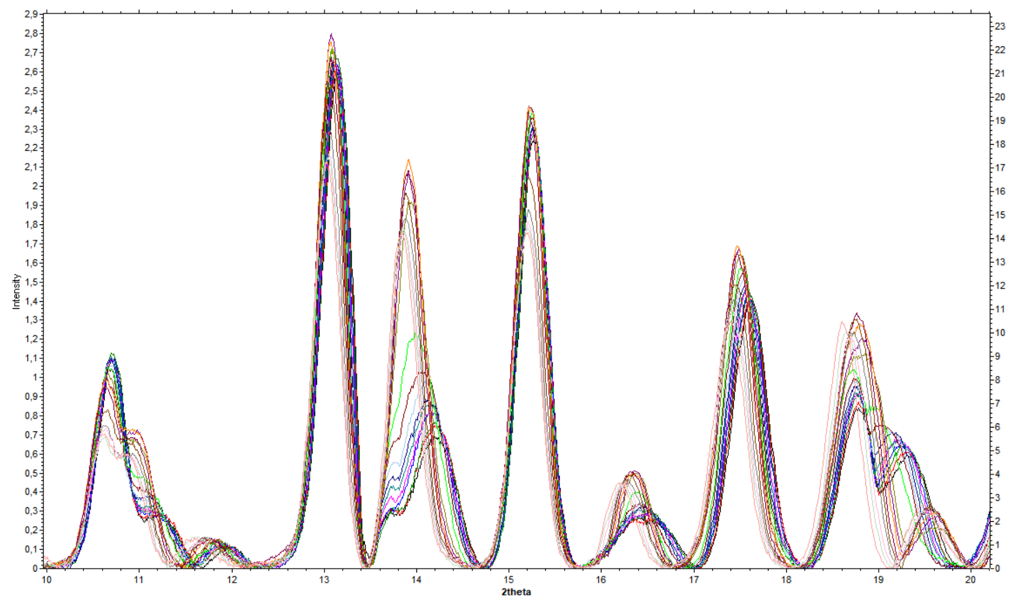


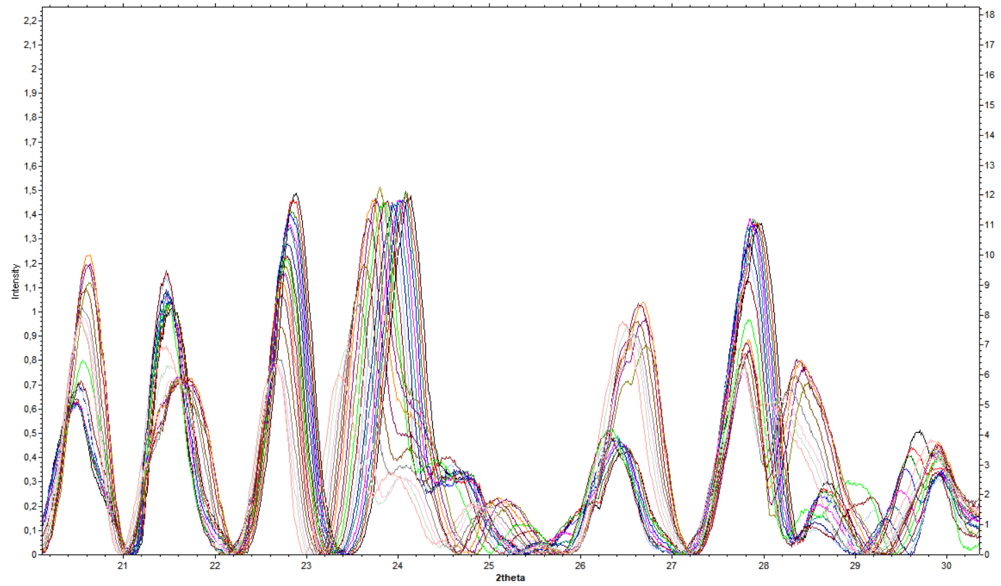

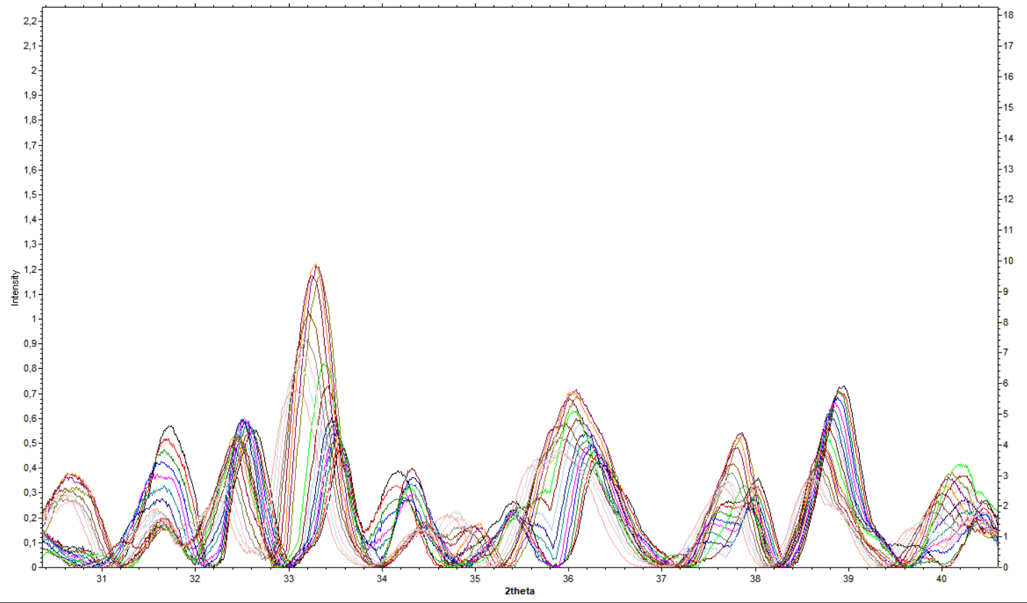


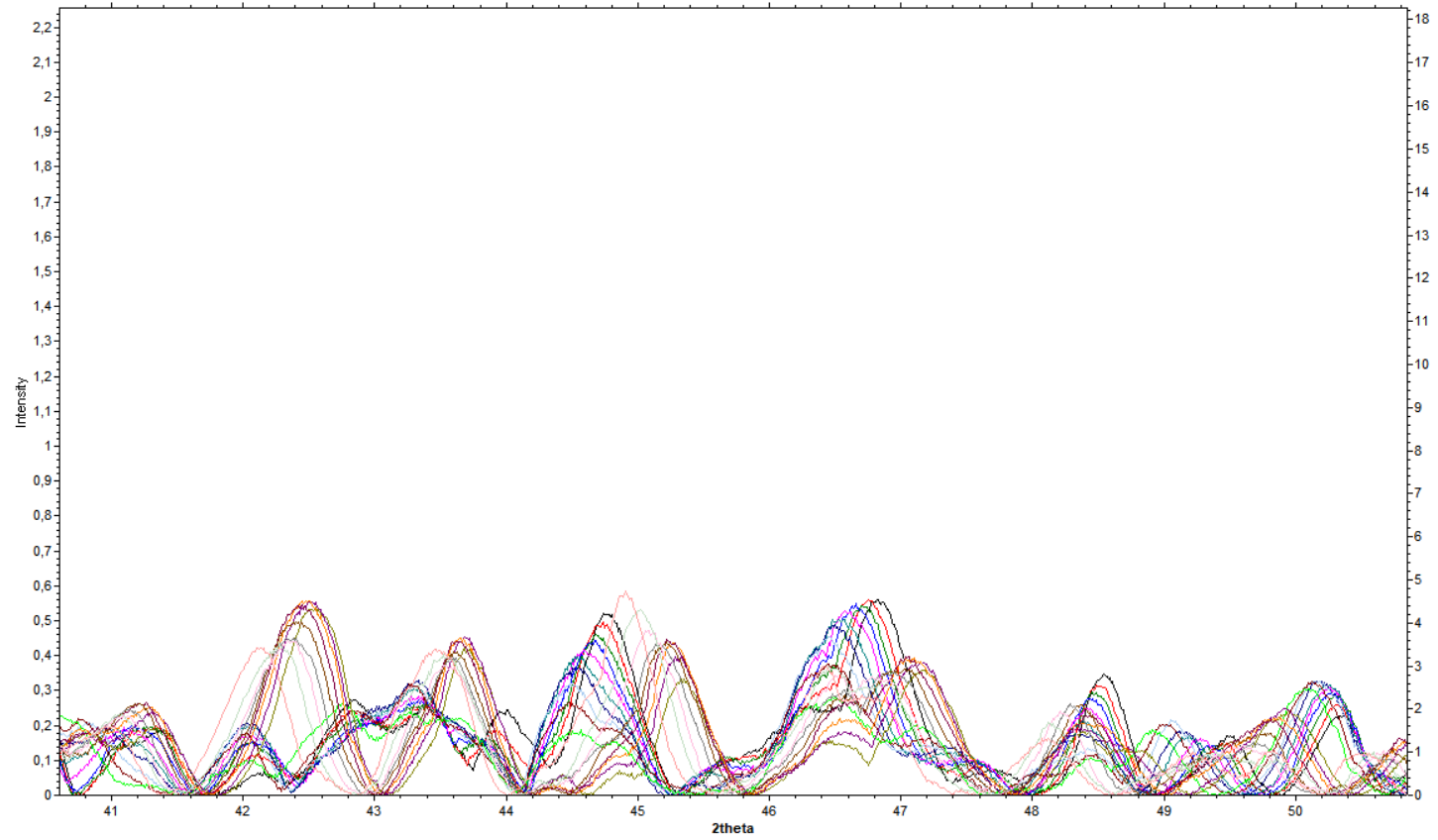


**Figure S12.** Fragments of the temperature PXRD patterns for compound **2** at different temperatures

|  |  |
| --- | --- |
|  |  |

**Fig. S9.** The graphs presenting the percentage of specific contacts in complex 1 (green) and 2 (red)
